# Ligand-dependent differences in estrogen receptor beta-interacting proteins identified in lung adenocarcinoma cells corresponds to estrogenic responses

**DOI:** 10.1186/1477-5956-9-60

**Published:** 2011-09-27

**Authors:** MM Ivanova, SM Abner, WM Pierce, CM Klinge

**Affiliations:** 1 Department of Biochemistry & Molecular Biology, Center for Genetics and Molecular Medicine, University of Louisville School of Medicine, Louisville, KY. 40292 USA; 2 Department of Pharmacology and Toxicology, Center for Genetics and Molecular Medicine, University of Louisville School of Medicine, Louisville, KY. 40292 USA

## Abstract

**Background:**

A recent epidemiological study demonstrated a reduced risk of lung cancer mortality in breast cancer patients using antiestrogens. These and other data implicate a role for estrogens in lung cancer, particularly nonsmall cell lung cancer (NSCLC). Approximately 61% of human NSCLC tumors express nuclear estrogen receptor β (ERβ); however, the role of ERβ and estrogens in NSCLC is likely to be multifactorial. Here we tested the hypothesis that proteins interacting with ERβ in human lung adenocarcinoma cells that respond proliferatively to estradiol (E_2_) are distinct from those in non-E_2_-responsive cells.

**Methods:**

FLAG affinity purification of FLAG-ERβ-interacting proteins was used to isolate ERβ-interacting proteins in whole cell extracts from E_2 _proliferative H1793 and non-E_2_-proliferative A549 lung adenocarcinoma cell lines. Following trypsin digestion, proteins were identified using liquid chromatography electrospray ionization tandem mass spectrometry (LC-MS/MS). Proteomic data were analyzed using Ingenuity Pathway Analysis. Select results were confirmed by coimmunoprecipitation.

**Results:**

LC-MS/MS identified 27 non-redundant ERβ-interacting proteins. ERβ-interacting proteins included hsp70, hsp60, vimentin, histones and calmodulin. Ingenuity Pathway Analysis of the ERβ-interacting proteins revealed differences in molecular and functional networks between H1793 and A549 lung adenocarcinoma cells. Coimmunoprecipitation experiments in these and other lung adenocarcinoma cells confirmed that ERβ and EGFR interact in a gender-dependent manner and in response to E_2 _or EGF. BRCA1 interacted with ERβ in A549 cell lines and in human lung adenocarcinoma tumors, but not normal lung tissue.

**Conclusion:**

Our results identify specific differences in ERβ-interacting proteins in lung adenocarcinoma cells corresponding to ligand-dependent differences in estrogenic responses.

## Background

A recent epidemiological study reported reduced risk of lung cancer mortality in breast cancer patients using antiestrogens, suggesting further study is needed to examine the potential of antiestrogens to reduce lung cancer risk [1]. The role of estrogens in lung cancer initiation and disease progression remains unclear; however, estrogens are known to induce differentiation and maturation of normal lung tissue [2,3]. Some epidemiologic data indicate that women have a higher risk of lung adenocarcinoma, a type of non-small cell lung cancer (NSCLC), compared to men [4,5]. A positive correlation between post-menopausal estrogen replacement therapy, smoking, and lung adenocarcinoma was reported in one study [6]. The mechanisms underlying the apparent role of gender and estrogens in NSCLC is not yet understood [7]. Local estrogen production may play a role since NSCLC carcinomas had higher estradiol (E_2_) concentrations compared to the corresponding non-neoplastic lung tissues from the same patient, regardless of gender [8]. E_2 _concentrations correlated with aromatase (*CYP19A1*) mRNA, but not with estrogen receptor α or b (ERα or ERβ) staining [8]. E_2 _concentration was positively associated with tumor size and Ki-67 staining in ERβ-positive NSCLC tumors from male patients but not postmenopausal female patients [8]. Likewise, cytosolic ERβ was a prognostic indicator of reduced survival in male, but not female NSCLC tumors [9]. Aromatase and ERβ expression were correlated, reflecting a more differentiated and less invasive phenotype [10].

Estrogens may contribute to lung tumorigenesis through mechanisms involving genomic, membrane-initiated, and mitochondrial ER-regulated activities. ERs bind directly to estrogen response elements (EREs) or interact with other DNA-bound transcription factors, *e.g*., AP-1, Sp1, and NF-κB, via a "tethering mechanism" [11,12]. These interactions recruit coregulators and either activate or suppress gene transcription in a ligand- and gene- specific manner (reviewed in [13]). A second mechanism by which estrogens regulate cell function is by a membrane-initiated, 'pre-genomic' or 'nongenomic' signaling pathway involving activation of intracellular protein kinases, *e.g*., PI3K, MAPK, JNK, within minutes of treatment. These rapid signaling events are mediated through plasma membrane-associated ERα and/or GPR30/GPER [14] and involve cross-talk with other plasma membrane receptors, *e.g*., EGFR and IGF-R [12,15-17]. ERβ is in mitochondria of NSCLC cells [18-21]. ERβ interacts with proapoptotic Bad in a ligand-independent manner protecting NSCLC cells from apoptosis-inducing agents, *e.g*., cisplatin [20]. These data indicate that downregulating ERβ may be beneficial in NSCLC.

Both ERα and ERβ are expressed in normal lung tissue and in lung adenocarcinomas [18,21-25]. ERβ is the predominant ER subtype in adult human lung and ERβ expression is higher in lung adenocarcinoma than in normal lung tissue [26-28]. Interestingly, men with ERβ-positive tumors had a significant reduction in mortality compared with those with ERβ-negative tumors; whereas women with ERβ-positive tumors exhibited increased mortality [29]. Studies from our lab showed that E_2 _did not stimulate estrogenic responses, including proliferation, in normal lung bronchial epithelial cells [18], but stimulated proliferation of lung adenocarcinoma cell lines from females, but not males, through genomic ER regulation [22]. E_2 _had no effect on the intracellular distribution of ERβ and showed no gender difference [18]. Since the biochemical function of ERβ in lung adenocarcinoma is unknown, the identification of ERβ interacting proteins is essential to dissect ERβ's role in the lung cancer progression.

Since ERβ's discovery in 1996 [30], 47 proteins have been reported to interact with ERβ including DP97 DEAD-box RNA helicase [31], SHP [32], BCAS2 [33], the p160 coactivator SRC-1/NCOA1 [34], and other coregulators (reviewed in [13]),. Additional proteins that interact with ERβ in the cytoplasm including STAT 1, 3 and 5 [35,36], calmodulins 1, 2 and 3 [37], and AKT [38]. ERβ interacts with Bad in mitochondria [20]. Surprisingly, to the best of our knowledge, no one has analyzed ERβ-interacting proteins using a proteomics approach in NSCLC cells derived from female *versus *male patients.

The goal of the present study was to identify ERβ-interacting proteins in lung adenocarcinoma cells and how E_2 _affects the identity of ERβ-interacting proteins. Here we describe the identification of ERβ-interacting proteins using immunoaffinity precipitation followed by mass spectrometry analysis and characterization of ERβ-interacting proteins. Identification of ERβ-interacting proteins may lead to new understandings of the role of ERβ in lung cancer.

## Materials and methods

### Antibodies

Antibodies (ab) were purchased as follows: ERβ (H-150), EGFR (1005), and HDAC (H-51)from Santa Cruz Biotechnology; ERβ (06-629), calmodulin (05-173), and BRCA1 (07-434) from Millipore; FLAG, β-actin (ACTB) from Sigma, α-tubulin (Ab-2) and EGFR (Ab-13) from Thermo-Fisher Scientific.

### Cell lines and treatment

NCI-H1793, A549, NCI-H1792, and NCI-H1944 were purchased from ATCC and maintained as previously described [22]. Prior to treatment, cells were placed in phenol red-free media supplemented with 5% dextran-coated, charcoal-stripped FBS (DCC-FBS) for 72 h. Cells were treated with ethanol (EtOH, a vehicle control), 10 nM E_2_, 100 nM 4-OHT, 10 ng/ml EGF or combination for 1 h prior to harvest. Whole cell extracts (WCE) were prepared in NP-40 IP buffer containing 50 mM Tris, 150 mM NaCl, 0.5% NP-40, 1 mM EDTA and protease and phosphatase inhibitors added fresh prior to harvest.

### Sources of patient samples

8 samples of normal (N) or tumor (T) lung tissue from NSCLC patients were supplied by Fox Chase Cancer Center https://studies.fccc.edu. The gender distribution of the samples was 4 women and 4 men. The median age was 67.5 years for women and 69.5 years for men. NSCLC tumors were adenocarcinomas, stages 1 A or B with grade types poorly, moderate or well differentiated (Table 1).

**Table 1:** Characteristics of human lung tumor samples.

Samples Tumor	Specimen	Gender	Age	Race	Grade	Stage	Diagnosis
1	1001216	F	70	White	well differentiated	1A	Adenocarcinoma
2	1002745	F	56	White	poorly differentiated	1A	Adenocarcinoma
3	1002940	F	81	White	moderately differentiated	1B	Adenocarcinoma
4	1002800	F	63	White	poorly differentiated	1B	Adenocarcinoma
5	1003775	M	65	White	poorly differentiated	1A	Adenocarcinoma
6	1003735	M	68	White	moderately differentiated	1B	Adenocarcinoma
7	1001746	M	80	White	moderately differentiated	1	Adenocarcinoma
8	1004066	M	65	White	moderately differentiated	1B	Adenocarcinoma

Human lung tumor samples were purchased from Fox Chase Cancer Center.

### Affinity purification of rhFLAG-ERβ interacting proteins

1 mg of WCE from H1793 and A549 was preincubated with 355 fmol rhFLAG-ERβ [39] for 1 h at 4°C and then incubated with EZview™ Red ANTI- FLAG-M2 affinity beads (Sigma) overnight at 4°C with rotation. The beads were sedimented, rinsed with 500 μl of ice cold TBS buffer (50 mM Tris-HCl, pH 7.4; 150 mM NaCl, 1 mM EDTA, 0.5% NP-40) three times. FLAG-ERβ and its associated proteins were eluted with 6 M urea and identified by mass spectrometry (Additional file 1, Figure S1). For validation of the specificity and efficiency of ERβ interaction with ANTI- FLAG-M2 affinity beads, 10 μl of the eluted protein complex was resolved on 10% SDS gels and transferred to PVDF membranes that were probed with anti-ERβ H150 antibody (Santa Cruz) (Additional file 2, Figure S2). A band of ~ 60 kDa corresponding to the long form of ERβ1 was identified in the ethanol (EtOH, vehicle control) and E_2_- treated H1793- and A549-rhFLAG-ERβ pull-down lanes but not in the lanes without added FLAG-ERβ. A lower MW band in the A549 samples is nonspecific, perhaps IgG (Additional file 2, Figure S2). The efficiency of FLAG-ERβ elution was 79.4 ± 4.4% (Additional file 2, Figure S2).

### Protein Identification by LC-MS/MS

Protein samples from immunoprecipitation were dried by speedvac and dissolved with 8 M urea in 50 mM NH_4_HCO_3 _(pH 8). The samples were reduced with dithiothreitol, alkylated with iodoacetamide, diluted with 50 mM NH_4_HCO_3 _and digested with sequencing grade modified trypsin (Promega, Madison, WI) at 37°C overnight. The digests were desalted with C_18 _spin column (Pierce, Rockford, IL), concentrated by speedvac, loaded on to a C_18 _nanoAcquity UPLC Trap column (Waters, Milford, MA), and then peptides in the samples were separated with a C_18 _nanoAcquity UPLC capillary column (Waters) with an acetonitrile and 0.1% formic acid gradient by a nanoAcquity LC system from Waters. The eluted peptides were directed to a LTQ Orbitrap XL mass spectrometer (Thermo Fisher Scientific, San Jose, CA) via a Triversa Nanomate system from Advion Biosciences (Ithaca, NY) and MS/MS spectra of the peptides were acquired by data dependent scan with mass resolution of 100,000 and 7,500 in MS and MS/MS mode respectively. The database search was performed by Proteome Discoverer 1.2 from Thermo Fisher Scientific with Sequest algorithm and the most current version of SwissProt database (Feb 8, 2011). High confident peptide matches of at least two different peptides are required for positive protein identification and XCorr scores > 1.9, 2.3 and 2.6 were considered high confident peptide matches for charge state 2, 3, and 4 of precursor ions respectively.

### Protein pathway analysis

Proteomic data were analyzed using Ingenuity Pathway Analysis (IPA) http://www.ingenuity.com. Networks were generated using gene identifiers that were uploaded into IPA.

### Co-immunoprecipitation and western blot

300 μg of WCE, cytoplasmic or nuclear extracts (CE or NE) were preincubated with rhFLAG-ERβ and then added to EZview™ Red ANTI- FLAG-M2 affinity beads using immunoprecipitation protocol. For analysis of endogenous ERβ, 300 μg WCE or 100 μg of CE and NE were preincubated with ERβ ab (06-629) overnight at 4°C and then added to ChIP-grade Protein G agarose beads (Cell Signaling). Proteins were eluted with Laemelli buffer and boiled. 1/2 of the volume of the eluted proteins was separated on 10% SDS gels and transferred to PVDF membranes. 30 μg of the starting WCE, CE or NE served as an input control. Super Signal West Pico Chemiluminescent Substrate (Pierce) was used to detect protein bands on Kodak BioMaxML film or a Carestream Imager. Un-Scan-It 6.1 for Windows (Silk Scientific) was used to digitalize and analyze the relative amounts of protein, based on pixel density, in the film immunoblot bands. Carestream molecular imaging software was used to analyze digital images.

### Immunofluorescence Staining

The H1793, H1792, H1944 and A549 cells were grown on coverslips. Before fixation, the cells were incubated in phenol red-free media supplemented with 5% DCC-FBS for 72 h and treated with 10 nM E_2_, 10 ng/ml EGF or combination for 1 h. Cells were washed with PBS, fixed with cold methanol:acetone (1:1) for 5 min, and washed twice with cold PBS. After blocking with 1% goat serum and 0.3% Triton X-100 in PBS for 30 min, primary antibodies (anti-mouse EGFR Ab-13 and anti-rabbit ERβ (06-629) were added at a 1:300 and 1:1000 dilution, respectively, for a 1 h incubation. The secondary anti-mouse antibody was labeled with Zenon Alexa Fluor 488 (green color) and the secondary anti-rabbit antibody was labeled with Zenon Alexa Fluor 594 (red color), both from Molecular Probes. Cells were then incubated with ProLong Gold antifade reagent with 4',6-diamidino-2-phenylindole (Molecular Probes). Images were captured using a Zeiss Axiovert 200 inverted microscope with a 63× objective lens using AxioVision Release 4.3 software.

## Results and Discussion

### Identification of ERβ interacting proteins by LC-MS/MS mass spectrometry analysis

A functional proteomic approach, summarized in Additional file 1, Figure S1, was used to identify proteins interacting with ERβ in two representative lung adenocarcinoma cell lines: H1793 and A549, derived from a female and male patient respectively. In brief, H1793 and A549 cells were incubated in phenol red-free medium in 5% charcoal-stripped serum for 3 days and then treated with EtOH or 10 nM E_2 _for 1 h. Whole cell extracts (WCE) were incubated with partially purified, baculovirus-expressed recombinant FLAG-ERβ1. We acknowledge that additional ERβ-interacting proteins might have been identified if we had overexpressed FLAG-ERβ in the cells, treated the cells with EtOH *versus *E_2 _and done the IP from these transfected cells. Reasons that we did not do the experiment this way include differences in transfection efficiency between the two cell lines and a concern as to how ERβ overexpression would affect endogenous protein expression in the cell lines. The specificity of FLAG affinity capture and elution of the FLAG-ERβ protein was demonstrated by western blot (Additional file 2, Figure S2). The lower MW band recognized by the ERβ H150 antibody in the A549 WCE was non-specific.

**Figure 1:**
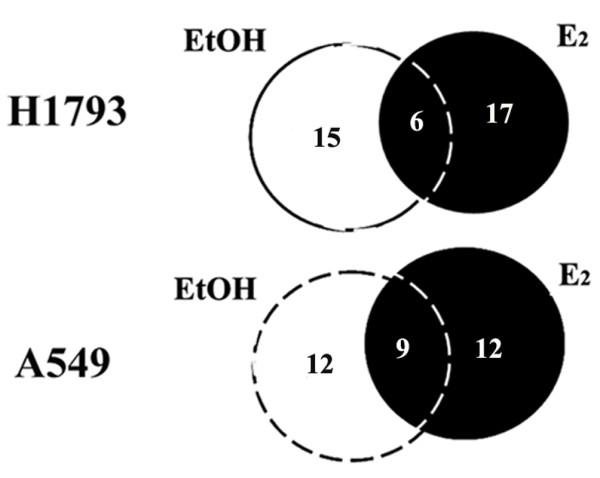
**Identification of ERβ-interacting proteins in lung adenocarcinoma cells**. The Venn Diagrams indicate number of the common and unique proteins identified by LC-MS/MS mass spectrometry in H1793 and A549 lung adenocarcinoma cell lines. The identity of these ERβ-interacting proteins is provided in Additional file 4, Table S2.

The eluted FLAG-ERβ-protein complexes were subjected to trypsin digestion followed by analysis by liquid chromatography tandem mass spectrometry (LC-MS/MS). Biological replicates were performed to assess reproducibility. A summary of the results is shown in Venn diagrams (Figure 1). Twenty-seven individual proteins interacting with ERβ were identified in WCE from A549 and H1793 cells (Additional file 3, Table S1). Recently, an LC-MS/MS approach identified 264 and 303 nuclear proteins associated with TAP-tagged ERα [40] and TAP-tagged ERβ [41] in MCF-7 breast cancer cells. We compared those data with our list of ERβ-associated proteins and found 6 common ERβ interacting proteins. We also found 9 proteins in our ERβ data set and that were previously reported to be ERα interacting proteins [40]. Common proteins to our ERβ interacting proteins data set and the ERα- and ERβ-associated proteins in MCF-7 cells include histones, calmodulin, hsp60, hsp70, β-actin (ACTB), and vimentin (Table 2). For EtOH- and E_2_- treated H1793 cells, 15 and 17 proteins were identified, respectively, with 6 proteins in common including hsp60 and histone H2A (Figure 1B, Table 2, Additional file 4, Table S2). For 4-OHT- treated H1793 cells, 10 proteins were identified, with 4 proteins in common with EtOH or E_2_-treated cells including hsp60, 40S ribosome, and tubulin. Unique 4-OHT/ERβ interacting proteins include γ-actin, 14-3-3ε protein and hsp90 (Additional file 5, Table S3).

**Table 2:** Identification of ERβ-interacting proteins in H1793 and A549 cells by LC-MS/MS.

	Cell line/treatment Protein name	gene name	HPRD		**Tarallo et al **[40]	
			**ERβ**	**ERα**	**ERβ**	**ERα**

	**H1793, EtOH**					

1	Tubulin beta-2A chain	TUBB2A				

2	Myosin-9	MYH9				+

3	β-actin	ACTB			+	+

4	Tubulin alpha-3C/D chain	TUBA3C				

5	Vimentin	VIM			+	

6	ERβ	ESR2	+		+	

7	Heat shock 70 kDa (Hsp70)	HSPA8		+	+	+

8	Histone H2A type 1-H	HIST1H2				

9	Heat shock 60 kDa (Hsp60)	HSPD1				+

10	Putative annexin A2-like protein	ANX2P2				

11	40S ribosomal protein S3	RPS3			+	

12	Protein arginine N-methyltransferase 5	PRMT5		+		

13	Calmodulin	CALM	+			+

14	Histone H4	HIST1H4A			+	

15	GTP-binding nuclear protein	RAN				

	**H1793, E_2_**					

1	β-actin	ACTB			+	+

2	Myosin-9	MYH9				+

3	Tubulin beta-2A chain	TUBB2A				

4	Tubulin alpha-3C/D chain	TUBA3C		+		

5	Tropomyosin alpha-4 chain	TPM3				

6	60 kDa heat shock protein	HSPD1				+

7	Histone H2A type 1-H	HIST1H2			+	

8	Heat shock 70 kDa protein 1-like	HSPA8		+	+	

9	Vimentin	VIM			+	

10	Nucleolin	NCL				+

11	Tropomyosin alpha-3 chain	TPM3				

12	Nucleophosmin	NPM1				+

13	Myosin-VI	MYO6				+

14	Plectin	PLEC				

15	40S ribosomal protein S3	RPC3			+	

16	60S ribosomal protein	RPL8				+

17	Heterogeneous nuclear ribonucleoproteins A2/B1	HNRNPA2				

	**A549, EtOH**					

1	Tubulin beta-2A chain	TUBB2A				

2	Actin	ACTB			+	

3	Tubulin alpha-3C/D chain	TUBA3C		+		

4	Heat shock 70 kDa protein	HSPA8		+	+	+

5	60 kDa heat shock protein	HSPD1				+

6	Histone H2A type 1-H	HIST1H2			+	

7	40S ribosomal protein S3	RPC3			+	

8	Histone H4	HIST1H2A			+	

9	Tropomyosin alpha-1 chain	TPM1				

10	Myosin-9	MYH9				

11	Calmodulin	CALM	+			

12	Myosin regulatory light chain 12A	MYL12A				

	**A549, E2**					

1	Tubulin beta-2A chain	TUBB2A				

2	Actin	ACTB			+	

3	Tubulin alpha-3C/D chain	TUBA3C		+		

4	Heat shock 70 kDa protein	HSPA8		+	+	

5	Myosin-9	MYH9				+

6	40S ribosomal protein S3	RPC3			+	

7	60 kDa heat shock protein	HSPD1				+

8	Histone H4	HIST1H2A			+	

9	Elongation factor 1-alpha 1	EEF1A1				+

10	Calmodulin	CALM	+			

11	Tropomyosin alpha-1 chain	TPM1				

12	40S ribosomal protein S23	RPC23				

H1793 and A549 cells were treated for 1 h with EtOH (vehicle) or 10 nM E_2 _and FLAG-ERβ-interacting proteins were immunocaptured, eluted, and trypsin-digested as described in Additional file 1, Figure S1. Trypsin-digested eluates were subjected to LC-MS/MS peptide identification (see Additional file 4, Table S2). Proteins are listed from highest to lowest score within each cell line/treatment group. The literature citations for previous identification of these proteins as interacting with ERβ and ERα are provided from the Human Protein Reference Database (HPRD, www.hprd.org) databases and Tarallo *et al*. [40].

For EtOH- and E_2_- treated A549 cells, 12 proteins were identified in each treatment with 9 proteins in common including tropomyosin, histone H4A, hsp60, and calmodulin (Figure 1B, Table 2, Additional file 6, Table S4). Five ERβ-interacting proteins, *i.e*., β-actin, hsp60, myosin9, RPS3, and tubulin beta-2, were detected in both H1793 and A549 cells with EtOH and E_2 _treatment (Additional file 6, Table S4). Interestingly, E_2 _stimulates hsp60 expression and hsp60 plays a role in mitochondrial protein import and macromolecular assembly [42]. Others have established a role for ERβ in mitochondrial function [43-46].

### Bioinformatic analysis of ERβ-interacting proteins

The proteomic data was analyzed using IPA to identify cellular distribution, canonical pathways, and functional groupings.

### Subcellular distribution of ERβ interacting proteins

**Figure 2:**
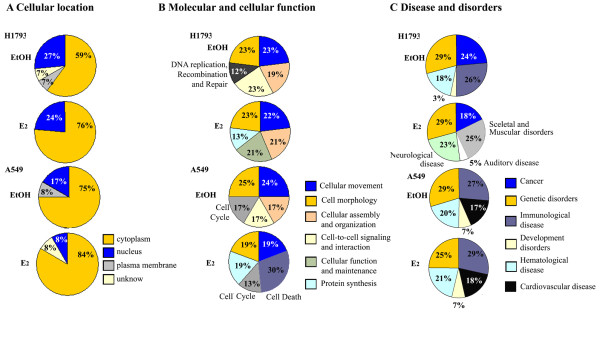
**Cellular location and molecular function of ERβ-interacting proteins**. The relative proportions of ERβ-interacting proteins identified in each cell line after EtOH or E_2 _treatment with LC-MS-MS with the subcellular localization (A), functional classification (**B**) and disease classification (**C**) was determined by IPA as described in the text.

First, the cellular localization of all identified ERβ-interacting proteins was examined using IPA (Figure 2A). IPA revealed most ERβ-interacting proteins are cytoplasmic (59-84%, respectively) with ~ 8-27% localized in the nucleus (Figure 2A). There is a clear distinction in subcellular localization in ERβ-interacting proteins between H1793 and A549 cells. More ERβ-interacting proteins were nuclear-localized in H1793 than in A549 cells. E_2 _increased the number of ERβ-interacting cytoplasmic proteins in both cell lines cells (Figure 2).

### Bioinformatic analysis of ERβ-interacting proteins

IPA was used to assign identified ERβ-interacting proteins into different molecular and functional classes based on biological evidence from the IPA literature database.

IPA of ERβ interacting proteins identified by LC-MS/MS revealed "cellular movement" and "cell morphology" as the most representative molecular functional classes in EtOH- and E_2_-treated H1793 and A549 cells (Figure 2B). In addition, the "cellular assembly and organization" functional class was noted in EtOH- and E_2_-treated H1793 cells and in the EtOH-treated A549 cells. Proteins in the "cell-to-cell signaling and interaction" functional class were detected in EtOH-treated cells. Interestingly, and in agreement with estrogen's roles in cellular functions in other cell types [47] and in these cell lines [18,21,22,39], in E_2_-treated H1793 and A549 cells, the functional class of cellular assembly and organization was noted (Figure 2B). The major differences in categorization of the ERβ-interacting proteins in H1793 *versus *A549 cells was the presence of the "cell cycle" class in EtOH- and E_2_- treated in A549 cells and "cell death" class in E_2_-treated A549 cells, but not in H1793 cells. Interestingly, the ERβ-interacting proteins from EtOH-treated H1793 cells were included in the "DNA replication, recombination and repair" class including MYH9 (organization of single fibers), VIM (morphology of nuclear matrix), and RAN (assembly nuclear envelope) proteins https://analysis.ingenuity.com/ (Figure 2B). In addition, MALDI-TOF mass spectrometry analyses (data not shown) identified another ERβ interacting protein with DNA repair function [48,49]: BRCA1 (4 sequenced peptides that match the full length protein, but with a low score 3.1. These DNA repair proteins (MYH9, VIM, RAN, and BRCA1) were selected for bioinformatic characterization (Additional file 7, Figure S3). IPA was performed on this protein set to identify associated functional network(s). The top representative function was cancer-related network with a score of 18.

As expected, IPA identified "cancer and genetic disorders related proteins" in the ERβ-interacting proteins (Figure 2C). Table 3 summarizes the IPA correlation of the identified ERβ-interacting proteins with cancer, including lung cancer. Notably, 13 proteins were linked to tumorigenesis, *e.g*., EEF1A1, hsp70, RAN, vimentin, and β-actin. The proteins associated with NSCLC include EEF1A1 and vimentin (Table 3).

**Table 3:** IPA-based functional category "cancer" correlates with the identified ERβ-interacting proteins.

IPA function	Total number of proteins identified	Protein				
			H1793	H1793	A549	A549
			EtOH	E_2_	EtOH	E_2_
cancer	12	ACTB,	+	+	+	+
		EEF1A1,				+
		ESR2,	+	+	+	+
		HSPA8,	+	+		
		MYL12A,			+	
		NCL		+		
		PLEC,		+		
		RAN,	+			
		TPM1,			+	+
		TPM3,		+		
		TUBB2A,	+	+	+	+
		VIM	+	+		

malignant tumor	7	EEF1A1,				+
		ESR2,	+	+	+	+
		HSPA8,	+	+		
		MYL12A,			+	
		NCL,		+		
		TUBB2A,	+	+	+	+
		VIM	+	+		

non-small-cell lung cancer	5	EEF1A1,				+
		ESR2,	+	+	+	+
		MYL12A,			+	
		TUBB2A,	+	+	+	+
		VIM	+	+		

metastasis	4	ACTB,	+	+	+	+
		ESR2,	+	+	+	+
		TUBB2A,	+	+	+	+
		VIM	+	+		

The ERβ-interacting proteins are from Supplemental Tables 2 and 3. The total number of proteins in the IPA classification are indicated followed by the protein name. The + means that protein was identified in the indicated cell line (H1792 or A549) after EtOH or E_2 _treatment.

IPA pathway analysis was used to group ERβ-interacting proteins detected by LC-MS/MS into functional networks to determine the cellular activities that may be regulated by ERβ in lung cancer cells. For proteins identified in the cellular assembly and organization network (with the top score of 56), the NFκB signaling pathway linked many of the ERβ-interacting proteins, including VIM, HSPD1 (hsp60), and HSPA1L (hsp70). The resulting network also covered several "branches" including a direct interaction of ERβ and nuclear proteins affecting chromatin structure and gene regulation including those identified by LC-MS/MS, *i.e*., nucleolin and histones (Table 2, Additional file 8, Figure S4).

Finally, IPA was used to identify the differences in functional networks of ERβ-interacting proteins between H1793 and A549 cells treated with EtOH or E_2_. For EtOH-treated H1793 cells, the top network (score 31) was "tissue development, cell morphology and genetic disorders" and the pathways were linked to ERK1/2 and NFκB signaling pathways (Additional file 9, Figure S5). For E_2_-treated H1793 cells, the top network was "cellular function and maintenance" (score 47) and the pathways were linked not only to NFκB and ERK1/2, but also to the FSH pathway (Additional file 9, Figure S5) by the ERβ-interacting proteins HSPD1 (hsp60), HSPA1L (hsp70) and tropomyosins (TPM4 and 3). Tropomyosins are involved in cell movement and act as interpreters of the local signaling environment in human cancer cells [50]. For EtOH-treated A549 cells, the top network of ERβ-interacting proteins was "cell-to-cell signaling and interaction" (score 34), which was linked to the FSH pathway by Ca^2+^, tropomyosin (TPM1), calmodulin (CALM), β-actin (ACTB) and transforming growth factor β 1 proteins (TGFβ1) (Additional file 10, Figure S6). For E_2_-treated A549 cells, the top network was "drug metabolism, endocrine system development and function" (score 31), which was linked first to FSH and steroid hormones pathways and secondarily to EGFR and TGFB1 (Additional file 10, Figure S6). Moreover, mass spectrometry identified EGFR in control- and E_2_- treated H1793 cells with 5 sequenced peptides that matched the full length EGFR, but with maximum score 4.4. These data indicate that ERβ cross-talks with the EGF signaling pathways by its interaction with EGFR, a result commensurate with a report that ERβ interacts with EGFR in human REN mesothelioma cells [51]. Additionally, ERα interacts with EGFR in MCF-7 breast cancer cells [52]. The mechanism of EGFR-ER cross-talk involves ERK1/2 activation, resulting phosphorylation of ser105 ERβ which plays an important role in its ligand-independent activation, nuclear localization, and transcriptional activity [53].

### Validation of MS/MS Data by Western blotting and Reciprocal Immunoprecipitation

**Figure 3:**
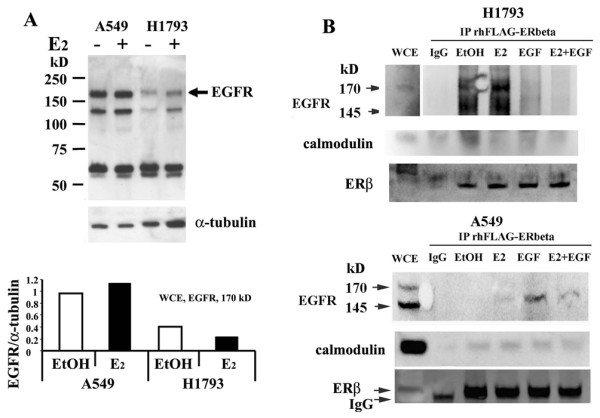
**Validation of ERβ-interacting proteins by coimmunoprecipitation and western blot**. **A**: H1793 and A549 cells were treated with 10 nM E_2 _for 1 h, whole cell extracts (WCE) were prepared and equal amts (30 μg protein) western blotted for EGFR. Higher EGFR protein expression was detected in A549 than H1793 cells. The arrow indicates the 170 kDa wild type EGFR. The membranes were stripped and reprobed for α-tubulin for normalization. The graph shows the quantification of EGFR expression. **B: **H1793 and A549 cells were treated with 10 nM E_2_, 10 ng/ml EGF, or the combination for 1 h. WCE of H1793 and A549 were incubated with rhERβ and then immunoprecipitated using a FLAG affinity beads or mouse IgG control and immunobloted using EGFR, calmodulin, and ERβ antibodies. EGFR bands were identified in the IP of H1793 cells treated with EtOH and E_2_, but not EGF or the combination of E_2 _and EGF treated.

Expression of select FLAG-ERβ1-interacting proteins identified in mass spectrometry, were first examined by Western blot analysis in each cell line. Because EGFR overexpression and mutations are linked to aggressive tumor biology including therapeutic resistance and poor clinical outcome in NSCLC [54,55] and since EGFR was previously reported to interact with ERβ [51] and ERα[56], we performed western and immunoprecipitation (IP) assays to examine ERβ-EGFR interaction. EGFR protein expression was higher in A549 than H1793 cells and A549 expresses both the 170 kDa (wild type) and a 145 kDa (vIII variant) of EGFR (Figure 3A). The 145 kDa EGFR (Δ 2-7) is a constitutively activated protein that is located in the plasma membrane and cytoplasm, is not regulated by EGF [57] and was reported to enhance the malignant phenotype [58,59]. Incubation of FLAG-ERβ1 with WCE followed by IP with FLAG affinity beads showed interaction of ERβ with 170 kD EGFR in both control and E_2_- treated samples in H1793 but not in A549 cell lines (Figure 3B). EGF blocked ERβ-EGFR interaction and E_2 _did not rescue this inhibition in H1793 cells (Figure 3B). Surprisingly, when A549 cells treated with EGF were IP'ed with FLAG affinity beads and ERβ, we observed EGFR-ERβ interaction and E_2 _blocked this interaction (Figure 3B). These results are commensurate with a previous report that EGF increased ERβ-EGFR interaction and E_2 _blocked ERβ-EGFR interaction in REN mesothelioma cells [51].

MS/MS analysis identified calmodulin (CALM) interaction with FLAG-ERβ in the EtOH-treated H1793 cells and EtOH- and E_2_-treated A549 cells (Table 2). Because CALM was reported to interact selectively with ERα and not ERβ[37] and with EGFR [60], we evaluated interaction of ERβ with CALM. Co-IP/western analysis confirmed ERβ-CALM interaction in the EtOH- and E_2_- treated H1793 cells and ligand independent ERβ-CALM interaction in A549 cell line (Figure 3B). These data provide the first evidence that ERβ interacts with CALM. Previous studies established that CALM directly interacts with ERα but not ERβ and the lack of interaction of ERβ with CALM was reported to be caused by a lack of CALM binding site conservation in ERβ [37]. Taken together, these results may be interpreted as indicating a non-direct interaction between ERβ and CALM. One possible explanation for our results is that ERα/ERβ heterodimers may interact with CALM via ERα-CALM interaction. Since H1793 and A549 express ERα and ERβ, it is likely that ERα/ERβ heterodimers exist in both cell lines. An alternative explanation is that the interaction may be indirect, for example, known CALM-interacting proteins include EGFR, myosin, and DDX5 http://www.hprd.org/ that also interact with ERβ, thus providing potential 'bridging partners'.

### Interaction of endogenous ERβ with EGFR

**Figure 4:**
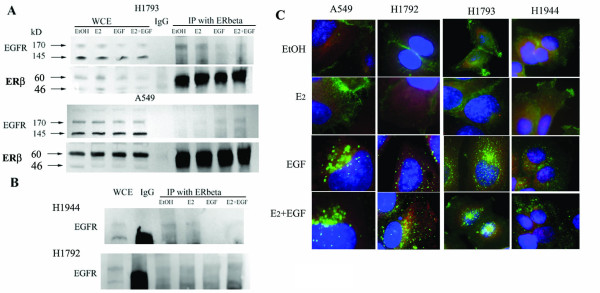
**Interaction of endogenous ERβ with EGFR**. A and B: H1793, A549, H1944 and H1792 cells were treated with 10 nM E_2_, 10 ng/ml EGF, or the combination for 1 h. 300 μg WCE were incubated with rabbit polyclonal ERβ antibody (Millipore) and them immunoprecipitate using a Protein G beads (Cell Signaling) or rabbit IgG control, and immunobloted using ab EGFR (1005). **A: **EGFR bands were identified in the IP of cytoplasmic (CE) and nuclear (NE) extracts of H1793 cells treated with EtOH and E_2_, but not EGF or the combination of E_2 _and EGF treated H1793 cell. EGFR bands were identified in the IP of cytoplasmic (CE) extracts of A549 cells treated with EGF or the combination of E_2_, but not EtOH and E_2 _treated cells. **B**: EGFR was identified in the ERβ IP from H1944 cells treated with EtOH and E_2_, but not EGF or the combination of E_2 _and EGF. EGFR bands were not identified in the IP from male derived H1792 cells. The membranes were stripped and reprobed for ERβ. **C: **The subcellular localization of EGFR and ERβ in lung adenocarcinoma cells was examined by immunofluorescent staining. The merged images are shown with anti-mouse EGFR Ab-13 (*green*) and anti-rabbit ERβ ab (06-629) (*red*) and counterstaining with DAPI (*blue*) in EtOH- and E_2_, EGF, E_2_+EGF treated H1793, H1792, H1944 and A549 cell lines. The individual images for each staining in Additional figure 11, Figure S7.

Because we identified proteins by interaction with baculovirus-expressed FLAG-ERβ protein, the next logical step was to confirm interaction of endogenous ERβ with the same proteins. Immunoprecipitation of WCE from H1793 and A549 cells with ERβ antibody (Millipore) detected ligand-dependent interaction of endogenous ERβ with EGFR in H1793 and A549 cell lines (Figure 4A and 4B). EGFR interacted with endogenous ERβ in H1793 cells treated with either EtOH or E_2_. EGF blocked EGFR-ERβ interaction and E_2 _did not affect the inhibition of EGFR-ERβ interaction seen with EGF treatment (Figure 4A). As seen for FLAG-ERβ in the co-IP studies, endogenous ERβ-EGFR interaction was not detected in the EtOH- and E_2_- treated A549 cells (Figure 4B). However, EGFR was co-IP'ed with endogenous ERβ in A549 cells treated with EGF or EGF plus E_2 _(Figure 4B). The molecular mechanism underlying these differences is unknown, but likely depends on cell-specific proteins that interact with both ERβ and EGFR. We were unable to perform the control blot for ERβ since IgG and ERβ have similar MWs. To test if ERβ interacts with EGFR in other lung adenocarcinoma cell lines, IP studies were performed using WCE from H1944 and H1792 lung adenocarcinoma cell lines from a female and male patient respectively (Figure 4B). Immunoprecipitation of ERβ in WCE from H1944 cells showed a pattern similar to that seen in H1793 cell lines: EGFR interacted with ERβ in the EtOH- and E_2_- treated H1944 cells and EGF blocked EGFR-ERβ interaction (Figure 4B). ERβ-EGFR interaction was not detected in H1792 cells (Figure 4B). We conclude that the gender of the patient from whom the lung adenocarcinoma cell line was derived correlate with endogenous ERβ interaction with EGFR, although there is some suggestion of a male-bias toward ERβ-EGFR interaction. Further studies with more samples and primary tumors will be required to verify any gender-dependence.

### Subcellular localization of EGFR and ERβ in lung adenocarcinoma cells

To further examine endogenous ERβ-EGFR interaction, and to assess whether subcellular localization is important in ligand-dependent interaction between ERβ and EGFR detected in co-IP studies, we performed immunofluorescent staining for ERβ and EGFR in EtOH-treated cells or in cells treated with E_2_, EGF, or both E_2 _and EGF for 1 h (Figure 4C, Additional file 11, Figure S7). First, we observed cell line-dependent differences in EGFR cellular localization between EtOH- and E_2_- treated cell lines derived from male (A549 and H1792) *versus *from female (H1793 and H1944) patients (Figure 4C). In EtOH- and E_2_- treated A549 and H1792 cells, EGFR was predominantly localized to the plasma membrane junction between cells and ERβ was cytoplasmic. In EtOH- and E_2_- treated H1793 and H1944 cell lines, EGFR showed plasma membrane localization, but also showed cytoplasmic and nuclear localization. These observations provide an explanation for the differences between ERβ/EGFR interaction in EtOH- and E_2_- treated male *versus *female derived cell lines. Surprisingly, EGF treatment resulted in a dynamic migration of EGFR into the cytoplasm and nucleus for all cell lines (Figure 4C). Although EGFR is a plasma membrane-bound receptor, a number of recent reports have validated nuclear EGFR localization and suggest a potential role the nuclear EGFR in tumor response to therapy [55]. For example, nuclear EGFR contributed to resistance to cetuximab in cancer cells including NSCLC [61]. To our knowledge, an association between gender differences and nuclear EGFR in lung adenocarcinoma is unknown. Women with lung adenocarcinoma are more sensitive to Gefitinib therapy and have greater overall survival than men because EGFR mutations are more prevalent in females [62]. Constitutively active EGFR mutants, *e.g*., L837Q and L723-P729insS, in NSCLC display cell-surface clustering even in the absence of EGF and are internalized from the cell surface [63]. Precisely how gender affects intracellular dynamics of EGFR, whether wildtype or mutant, following ligand-activation of EGFR is unknown and is the topic of ongoing investigation.

### Interaction of endogenous ERβ with BRCA1

**Figure 5:**
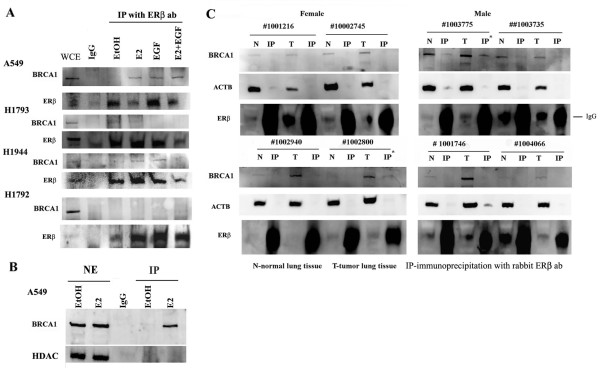
**Interaction of the endogenous ERβ with BRCA1**. A: A549, H1793 and H1792 cells were treated with 10 nM E_2_, 10 ng/ml EGF, or the combination for 1 h. 300 μg WCE were incubated with rabbit polyclonal ERβ antibody (Millipore), immunoprecipitated using a Protein G beads or rabbit IgG (negative control), and immunobloted using BRCA1 ab. The membranes were stripped and reprobed for ERβ. BRCA1 bands were identified in the IP from E_2_-, EGF-, E_2_+EGF- treated A549 cells and E_2_- and EGF- treated 1944 cells, but not in H1793 and H1792 cell. **B: **A549 cells were treated with 10 nM E_2 _for 1 h. 300 μg NE were incubated with ERβ (H150) ab, immunoprecipitated, and immunobloted using BRCA1 ab. BRCA1 bands were identified in the NE from E_2_-treated A549 cells. **C: **300 μg WCE from normal human lung tissue (N) or tumor human lung tissue (T) were incubated with ERβ (H150) ab, immunoprecipitated, and immunoblotted with BRCA1 ab. BRCA1 bands were identified only in the IP from two tumor samples from patients #1002800 and 1003775. The membranes were stripped and reprobed with β-actin (ACTB) as a loading control and then stripped and re-probed for ERβ. ERβ bands were identified in the WCE from normal or tumor lung tissue. IgG bands overlapped with ERβ in IP samples.

Several ERβ-associated proteins were found in the DNA repair function/network identified by IPA suggesting that DNA-bound ERβ may be involved in DNA repair, *e.g*., transcription-coupled DNA repair [64,65]. Because BRCA1 interacts directly with ERα and forms a complex between ERα and CBP that inhibits E_2_-stimulated ERα activity [66], we further investigated the possible BRCA1-ERβ interaction. The BRCA1 interaction site with ERα is LBD/AF2 region (aa 282-420) [67]. ERβ contains LBD/AF2 domain within 63% identities/87% positives to ERα protein, indicating the possibility of enough sequence/conformation within the LBD of the two subtypes for BRCA1 interaction. Further, low levels of BRCA1 have been reported in women with NSCLC [68]. Co-IP experiments showed that BRCA1 interacted with endogenous ERβ in E_2-_, EGF- and E_2_/EGF- treated A549 and in E_2_- and EGF- treated H1944 cells, but not in H1793 or H1792 cells (Figure 5A). Nuclear BRCA1 has been reported play a variety of roles including DNA repair, regulation of gene transcription, cell growth and apoptosis [69,70]. Western blot analysis of NE confirmed nuclear localization of BRCA1 in EtOH- and E_2_- treated A549 cell lines and BRCA1 was co-immunoprecipitated with ERβ in E_2_- treated A549 cells (Figure 5B). Future studies will examine if the E_2_-stimulated ERβ-BRCA1 interaction mediates estrogenic responses in A549 cells.

To provide translational relevance to our studies, we examined the interaction of ERβ with BRCA1 in 8 human lung adenocarcinomas (Figure 5C and Table 1). BRCA1 was immunoprecipitated with endogenous ERβ in tumor samples # 1002800 and #1003775 (Figure 5C and Table 1). Both tumors were poorly differentiated, one from a male and another from a female NSCLC patient. Importantly, ERβ-BRCA1 interaction was not detected in normal lung tissue from the same patients (# 1002800 and #1003775) showing ERβ/BRCA1 interaction in the tumor (Figure 5C). This suggests a 'gain of function' of BRCA1-ERβ interaction in the tumor. These data along with the IPA pathway analyses (Figure 2 and Additional file 7, Figure S3) suggest the potential ability of tumor suppressor BRCA1 to regulate the genomic ERβ signaling pathways in lung cancer, perhaps similar to BRCA1 function in breast cancer [71]. Further studies will be needed to assess the clinical significance of ERβ-BRCA1 interaction in NSCLC.

## Conclusions

In summary, these studies identified 27 ERβ-interacting proteins in two lung adenocarcinoma cell lines: H1793 and A549, and demonstrated cell- and ligand- specific differences in protein-ERβ interaction. Notably, IPA analysis identified 12 of the ERβ-interacting proteins as having roles in cancer progression and metastasis with 4 of these proteins having established roles in NSCLC, *i.e*., EEFIA, MYL12A, TUBB2A, VIM1 (Table 3). IPA analysis revealed that the proteins identified as interacting with ERβ are involved in cell movement, cell morphology, cellular assembly and organization, cell cycle and death, protein synthesis, and DNA replication, recombination and repair. The top network identified was "tissue development, cell morphology and genetic disorders". This functional network is linked by nongenomic/membrane-initiated ER signaling pathways with NFκB, ERK1/2, TGFB1, and EGFR signaling pathways and with the traditional genomic ER pathway. IPA identified EGFR as a part of the "drug metabolism, endocrine system development and function network" for ERβ-interacting proteins identified in our FLAG-ERβ pulldown. We confirmed that endogenous ERβ and EGFR interact and that E_2 _and EGF differentially modulate ERβ and EGFR interaction and subcellular distribution in a ligand- and cell line-dependent manner. Further, we identified BRCA1 as an endogenous ERβ-interacting protein in lung adenocarcinoma cell lines and in human lung adenocarcinomas. Further studies will be required to determine the precise role of these ERβ-interacting proteins as therapeutic targets or biomarkers in lung adenocarcinoma.

## List of Abbreviations

(4-OHT): 4-hydroxytamoxifen; (HAc): acetic acid; (ACN): acetonitrile; (ab): antibodies; (CALM): calmodulin; (CE): cytoplasmic extract; (DCC-FBS): 5% dextran-coated, charcoal-stripped fetal bovine serum; (DBD): DNA binding domain; (EGF): epidermal growth factor; (EGFR): epidermal growth factor receptor; (E_2_): estradiol; (ERα): estrogen receptor α; (ERβ): estrogen receptor β; (P-ser118-ERα): serine-118-phospho-ERα; (αERKO and βERKO, respectively): mice in which ERα and/or ERβ were deleted; (FBS): fetal bovine serum; (EtOH, vehicle control): ethanol; (ICI 182,780): faslodex/fulvestrant; (IP): immunoprecipitation; (IPA): Ingenuity Pathway Analysis; (LBD): ligand binding domain; (LC-MS/MS): liquid chromatography electrospray ionization tandem mass spectrometry; (MALDI-TOF-MS): matrix-assisted laser desorption/ionization mass spectrometry; (MW): molecular weight; (N): normal; (NE): nuclear extract; (NSCLC): nonsmall cell lung cancer; (T): tumor; (WCE): whole cell extracts;

## Competing interests

The authors declare that they have no competing interests.

## Authors' contributions

MMI performed the cell-based studies, IPA, prepared the Figures and contributed to the writing of the text; SMA performed co-IP experiments; WMP, Jr. performed all LC-MS/MS and peptide identification; CMK participated in all data analysis, Figure preparation, and writing of the manuscript. All authors read and approved the manuscript.

## Supplementary Material

Additional file 1 **Supplemental Figure 1: Experimental design for identification of ERβ-FLAG interacting proteins in transfected A549 and H1793 lung adenocarcinoma cells**. A549 and H1793 cells were incubated in phenol red-free medium with 5% DCC-stripped serum for 3 days prior to 1 h treatment with ethanol (EtOH, 1:1,000 dilution) or 10 nM E_2_. WCE (1 mg) from H1793 and A549 was preincubated with or without 355 fmol rhFLAG-ERβ for 1 h at 4°C and added to EZview™Red ANTI- FLAG-M2 affinity beads (Sigma) followed by overnight incubation at 4°C with rotation. After rinsing, as indicated, proteins were eluted with 6 M urea and digested with trypsin prior to LC-MS/MS analysis described in Materials and Methods. In parallel, samples of eluted proteins were separated by SDS PAGE gels and were stained with silver or were transferred for western blot. These western blot images demonstrate ERβ-protein capture. Ingenuity Pathway Analysis (IPA) was used to identify defined canonical pathways and functional classifications of the identified ERβ-interacting proteins. Click here for file

Additional file 2 **Supplemental Figure 2: Confirmation of the immunoprecipitation of ERβ**. WCE prepared from EtOH or E2- treated H1793 and A549 cells were incubated with FLAG-ERβ as described in Materials and Methods. FLAG-ERβ and interacting proteins were immunoprecipitated using Anti-FLAG M2 affinity beads (Lanes 1-4) and after elution 10 μl of the 100 μl samples was loaded. As a negative control, WCE were incubated with the FLAG beads (Lanes 5-6). Lane 7 was 35.5 fmol rhFLAG-ERβ. The blot was probed with ERβ (H150) antibody. A band at 59 kDa corresponding to ERβ was identified in the IP of H1793 and A549 cell lysates incubated with purified rhFLAG-ERβ protein but not in H1793 or A549 cell extracts incubated with FLAG beads without added rhFLAG-ERβ protein, demonstrating the specificity of the immunocapture for FLAG-ERβ. A nonspecific band of 50 kDa (NS) that was recognized by the ERβ antibody was bound by the FLAG beads in the A549 cells. This may be a splice variant of ERβ. The efficiency of eluting rh-FLAG-ERβ from beads was evaluated by counting the integrated optical densities (IOD) by Un-Scan-It (Silk Scientific, Orem, UT, USA). IODs bands of interest were divided to the control 35.5 fmol rhFLAG-ERβ and counted as %. Click here for file

Additional file 3 **Supplemental Table 1: List of all ERβ interacting proteins identified in ANTI- FLAG-M2 affinity beads eluates**. Shown are SwissProt ID, protein synonyms and abbreviation, gene name, subcellular location, molecular function types, biomarker applications, entrez human ID. Click here for file

Additional file 4 **Supplemental Table 2: Identification of ERβ interacting proteins in H1793 and A549 cells by LC-MS/MS**. This table lists proteins identified as interacting with ERβ in H1793 and A549 lung adenocarcinoma cells treated with EtOH or E_2_. Click here for file

Additional file 5 **Supplemental Table 3: Identification of ERβ-interacting proteins in 4-hydroxytamoxifen (4-OHT) treated H1793 by LC-MS/MS**. This table lists proteins identified as interacting with ERβ in H1793 lung adenocarcinoma cells treated with 100 nM 4-OHT. Click here for file

Additional file 6 **Supplemental Table 4: List of common ERβ interacting proteins identified in ANTI- FLAG-M2 affinity beads eluates**. This table lists proteins identified as interacting with ERβ in H1793 and A549 lung adenocarcinoma cells treated with EtOH and E_2 _or in both H1793 and A549 cells treated with EtOH and E_2_. Click here for file

Additional file 7 **Supplemental Figure 3: "DNA replication, recombination and repair" network of ERβ-interacting proteins identified in LS-MS/MS**. Proteins shaded in grey were identified as ERβ-interacting proteins. Proteins in white are those identified by Ingenuity Knowledge Base. The shapes denote the molecular class of the protein (◇enzyme, ▬ ligand-dependent nuclear receptor, ● other, double circle-group, hexagone-translational regulator). Solid lines indicate direct molecular interaction and dashed lines indicate indirect molecular interaction. Click here for file

Additional file 8 **Supplemental Figure 4: Network pathway analysis of total ERβ-interacting proteins identified in LS-MS/MS**. Proteins shaded in grey were identified as ERβ-interacting proteins. Proteins in white are those identified by Ingenuity Knowledge Base. The shapes denote the molecular class of the protein (◇enzyme, ▬ ligand-dependent nuclear receptor, ● other, double circle-group, hexagone-translational regulator). Solid lines indicate direct molecular interaction and dashed lines indicate indirect molecular interaction. Click here for file

Additional file 9 **Supplemental Figure 5: Network pathway analysis of ERβ-interacting proteins in EtOH-(A) and E_2_- (B) treated H1793 cell lines identified by LC-MS/MS**. Proteins shaded in grey were identified as ERβ-interacting proteins. Proteins in white are those identified by Ingenuity Knowledge Base. The shapes denote the molecular class of the protein (◇enzyme, ▬ ligand-dependent nuclear receptor, ● other, double circle-group, hexagone-translational regulator) (Table 2). Solid lines indicate direct molecular interaction, dashed lines indicate indirect molecular interaction and blue lines indicate the proteins discussed in the text. Click here for file

Additional file 10 **Supplemental Figure 6: Network pathway analysis of ERβ-interacting proteins in EtOH (A) and E_2 _(B) treated A549 cell lines identified by LC-MS/MS**. Proteins shaded in grey were identified as ERβ-interacting proteins. Proteins in white are those identified by Ingenuity Knowledge Base. The shapes denote the molecular class of the protein (◇enzyme, ▬ ligand-dependent nuclear receptor, ● other, double circle-group, hexagone-translational regulator) (Table 2). Solid lines indicate direct molecular interaction, dashed lines indicate indirect molecular interaction and blue lines indicate the proteins discussed in the text. Click here for file

Additional file 11 **Supplemental Figure 7: Subcellular localization of EGFR and ERβ in lung adenocarcinoma cells**. The indicated lung adenocarcinoma cell lines were treated with EtOH, E_2_, EGF, or E_2_+EGF- for 6 h. Merged images for EGFR and ERβ immunocytochemical staining are shown with anti-mouse EGFR Ab-13 (*green*) and anti-rabbit ERβ ab (06-629) (*red*). Cells were counterstained with DAPI (*blue*). At the far right of each panel are non-merged images for EGFR (green) and ERβ (red). Dotted lines outline the cell areas enlarged in the middle panel. Click here for file

## References

[B1] Bouchardy C Benhamou S Schaffar R Verkooijen HM Fioretta G Schubert H Vinh-Hung V Soria J-C Vlastos G Rapiti E Lung cancer mortality risk among breast cancer patients treated with anti-estrogens Cancer 2011 117 1288 1295 10.1002/cncr.25638 21264820

[B2] Dubey S Siegfried JM Traynor AM Non-small-cell lung cancer and breast carcinoma: chemotherapy and beyond The Lancet Oncology 2006 7 416 424 10.1016/S1470-2045(06)70693-3 16648046

[B3] Pietras RJ Marquez DC Chen HW Tsai E Weinberg O Fishbein M Estrogen and growth factor receptor interactions in human breast and non-small cell lung cancer cells Steroids 2005 70 372 381 10.1016/j.steroids.2005.02.017 15862820

[B4] Ramchandran K Patel JD Sex Differences in Susceptibility to Carcinogens Seminars in Oncology 2009 36 516 523 10.1053/j.seminoncol.2009.09.005 19995643

[B5] Kiyohara C Ohno Y Sex differences in lung cancer susceptibility: A review Gender Medicine 2010 7 381 401 10.1016/j.genm.2010.10.002 21056866

[B6] Taioli E Wynder EL Re: Endocrine factors and adenocarcinoma of the lung in women J Natl Cancer Inst 1994 86 869 870 10.1093/jnci/86.11.869 8182770

[B7] Siegfried JM Hershberger PA Stabile LP Estrogen Receptor Signaling in Lung Cancer Seminars in Oncology 2009 36 524 531 10.1053/j.seminoncol.2009.10.004 19995644 PMC2818861

[B8] Niikawa H Suzuki T Miki Y Suzuki S Nagasaki S Akahira J Honma S Evans DB Hayashi S-i Kondo T Sasano H Intratumoral Estrogens and Estrogen Receptors in Human Non-Small Cell Lung Carcinoma Clin Cancer Res 2008 1078 0432 CCR-1007-1950 10.1158/1078-0432.CCR-07-1950 18579664

[B9] Stabile LP Dacic S Land SR Lenzner DE Dhir R Aquafondata M Landreneau RJ Grandis JR Siegfried JM Combined analysis of estrogen receptor β-1 and progesterone receptor expression identifies lung cancer patients with poor outcome Clinical Cancer Research 2011 10.1158/1078-0432.CCR-10-0992 PMC3064257 21062926

[B10] Taneja SS Ha S Swenson NK Huang HY Lee P Melamed J Shapiro E Garabedian MJ Logan SK Cell-specific Regulation of Androgen Receptor Phosphorylation in Vivo J Biol Chem 2005 280 40916 40924 10.1074/jbc.M508442200 16210317

[B11] Paech K Webb P Kuiper GG Nilsson S Gustafsson J Kushner PJ Scanlan TS Differential ligand activation of estrogen receptors ERalpha and ERbeta at AP1 sites Science 1997 277 1508 1510 10.1126/science.277.5331.1508 9278514

[B12] Heldring N Pike A Andersson S Matthews J Cheng G Hartman J Tujague M Strom A Treuter E Warner M Gustafsson JA Estrogen receptors: how do they signal and what are their targets Physiol Rev 2007 87 905 931 10.1152/physrev.00026.2006 17615392

[B13] Klinge CM Estrogen receptor interaction with co-activators and co-repressors Steroids 2000 65 227 251 10.1016/S0039-128X(99)00107-5 10751636

[B14] Prossnitz ER Maggiolini M Mechanisms of estrogen signaling and gene expression via GPR30 Molecular and Cellular Endocrinology 2009 308 32 38 10.1016/j.mce.2009.03.026 19464786 PMC2847286

[B15] Chambliss KL Shaul PW Estrogen modulation of endothelial nitric oxide synthase Endocr Rev 2002 23 665 686 10.1210/er.2001-0045 12372846

[B16] Levin ER Cell localization, physiology, and nongenomic actions of estrogen receptors J Appl Physiol 2001 91 1860 1867 11568173 10.1152/jappl.2001.91.4.1860

[B17] Levin ER G Protein-Coupled Receptor 30: Estrogen Receptor or Collaborator? Endocrinology 2009 150 1563 1565 10.1210/en.2008-1759 19307418 PMC2659267

[B18] Ivanova MM Mazhawidza W Dougherty SM Minna JD Klinge CM Activity and intracellular location of estrogen receptors [alpha] and [beta] in human bronchial epithelial cells Mol Cell Endocrinol 2009 205 12 21 10.1016/j.mce.2009.01.021 PMC2767333 19433257

[B19] Pedram A Razandi M Wallace DC Levin ER Functional Estrogen Receptors in the Mitochondria of Breast Cancer Cells Mol Biol Cell 2006 17 2125 2137 10.1091/mbc.E05-11-1013 16495339 PMC1446071

[B20] Zhang G Yanamala N Lathrop KL Zhang L Klein-Seetharaman J Srinivas H Ligand-Independent Antiapoptotic Function of Estrogen Receptor-{beta} in Lung Cancer Cells Mol Endocrinol 2010 24 1737 1747 10.1210/me.2010-0125 20660297 PMC2940472

[B21] Ivanova MM Mazhawidza W Dougherty SM Klinge CM Sex Differences in Estrogen Receptor Subcellular Location and Activity in Lung Adenocarcinoma Cells Am J Respir Cell Mol Biol 2010 42 320 330 10.1165/rcmb.2009-0059OC 19556604 PMC2830404

[B22] Dougherty SM Mazhawidza W Bohn AR Robinson KA Mattingly KA Blankenship KA Huff MO McGregor WG Klinge CM Gender difference in the activity but not expression of estrogen receptors alpha and beta in human lung adenocarcinoma cells Endocrine-Related Cancer 2006 13 113 134 10.1677/erc.1.01118 16601283 PMC1472635

[B23] Fasco MJ Hurteau GJ Spivack SD Gender-dependent expression of alpha and beta estrogen receptors in human nontumor and tumor lung tissue Mol Cell Endocrinol 2002 188 125 140 10.1016/S0303-7207(01)00750-X 11911952

[B24] Mollerup S Jorgensen K Berge G Haugen A Expression of estrogen receptors alpha and beta in human lung tissue and cell lines Lung Cancer 2002 37 153 159 10.1016/S0169-5002(02)00039-9 12140138

[B25] Canver CC Memoli VA Vanderveer PL Dingivan CA Mentzer RM Jr Sex hormone receptors in non-small-cell lung cancer in human beings J Thorac Cardiovasc Surg 1994 108 153 157 8028359

[B26] Omoto Y Kobayashi Y Nishida K Tsuchiya E Eguchi H Nakagawa K Ishikawa Y Yamori T Iwase H Fujii Y Expression, function, and clinical implications of the estrogen receptor beta in human lung cancers Biochem Biophys Res Commun 2001 285 340 347 10.1006/bbrc.2001.5158 11444848

[B27] Omoto Y Inoue S Ogawa S Toyama T Yamashita H Muramatsu M Kobayashi S Iwase H Clinical value of the wild-type estrogen receptor beta expression in breast cancer Cancer Lett 2001 163 207 212 10.1016/S0304-3835(00)00680-7 11165756

[B28] Raso MG Behrens C Herynk MH Liu S Prudkin L Ozburn NC Woods DM Tang X Mehran RJ Moran C Immunohistochemical Expression of Estrogen and Progesterone Receptors Identifies a Subset of NSCLCs and Correlates with EGFR Mutation Clinical Cancer Research 2009 15 5359 5368 10.1158/1078-0432.CCR-09-0033 19706809 PMC2893045

[B29] Schwartz AG Prysak GM Murphy V Lonardo F Pass H Schwartz J Brooks S Nuclear Estrogen Receptor {beta} in Lung Cancer: Expression and Survival Differences by Sex Clin Cancer Res 2005 11 7280 7287 10.1158/1078-0432.CCR-05-0498 16243798

[B30] Kuiper GG Enmark E Pelto-Huikko M Nilsson S Gustafsson J-A Cloning of a novel estrogen receptor expressed in rat prostate and ovary Proc Natl Acad Sci USA 1996 93 5925 5930 10.1073/pnas.93.12.5925 8650195 PMC39164

[B31] Rajendran RR Nye AC Frasor J Balsara RD Martini PG Katzenellenbogen BS Regulation of nuclear receptor transcriptional activity by a novel DEAD box RNA helicase (DP97) J Biol Chem 2003 278 4628 4638 10.1074/jbc.M210066200 12466272

[B32] Johansson L Bavner A Thomsen JS Farnegardh M Gustafsson JA Treuter E The orphan nuclear receptor SHP utilizes conserved LXXLL-related motifs for interactions with ligand-activated estrogen receptors Mol Cell Biol 2000 20 1124 1133 10.1128/MCB.20.4.1124-1133.2000 10648597 PMC85230

[B33] Qi C Zhu YT Chang J Yeldandi AV Rao MS Zhu YJ Potentiation of estrogen receptor transcriptional activity by breast cancer amplified sequence 2 Biochem Biophys Res Commun 2005 328 393 398 10.1016/j.bbrc.2004.12.187 15694360

[B34] Northrop JP Nguyen D Piplani S Olivan SE Kwan ST Go NF Hart CP Schatz PJ Selection of estrogen receptor beta- and thyroid hormone receptor beta-specific coactivator-mimetic peptides using recombinant peptide libraries Mol Endocrinol 2000 14 605 622 10.1210/me.14.5.605 10809226

[B35] Kotaja N Aittomaki S Silvennoinen O Palvimo JJ Janne OA ARIP3 (androgen receptor-interacting protein 3) and other PIAS (protein inhibitor of activated STAT) proteins differ in their ability to modulate steroid receptor-dependent transcriptional activation Mol Endocrinol 2000 14 1986 2000 10.1210/me.14.12.1986 11117529

[B36] Faulds MH Pettersson K Gustafsson JA Haldosen LA Cross-talk between ERs and signal transducer and activator of transcription 5 is E2 dependent and involves two functionally separate mechanisms Mol Endocrinol 2001 15 1929 1940 10.1210/me.15.11.1929 11682624

[B37] Garcia Pedrero JM Del Rio B Martinez-Campa C Muramatsu M Lazo PS Ramos S Calmodulin is a selective modulator of estrogen receptors Mol Endocrinol 2002 16 947 960 10.1210/me.16.5.947 11981030

[B38] Aquila S Sisci D Gentile M Middea E Catalano S Carpino A Rago V Ando S Estrogen receptor (ER)alpha and ER beta are both expressed in human ejaculated spermatozoa: evidence of their direct interaction with phosphatidylinositol-3-OH kinase/Akt pathway J Clin Endocrinol Metab 2004 89 1443 1451 10.1210/jc.2003-031681 15001646

[B39] Ivanova MM Mattingly KA Klinge CM Estrogen receptor beta yield from baculovirus lytic infection is higher than from stably transformed Sf21 cells Appl Microbiol Biotechnol 2007 74 1256 1263 10.1007/s00253-006-0784-9 17318543

[B40] Tarallo R Bamundo A Nassa G Nola E Paris O Ambrosino C Facchiano A Baumann M Nyman TA Weisz A Identification of proteins associated with ligand-activated estrogen receptor α in human breast cancer cell nuclei by tandem affinity purification and nano LC-MS/MS Proteomics 2011 11 172 179 10.1002/pmic.201000217 21182205

[B41] Nassa G Tarallo R Ambrosino C Bamundo A Ferraro L Paris O Ravo M Guzzi PH Cannataro M Baumann M A large set of estrogen receptor beta-interacting proteins identified by tandem affinity purification in hormone-responsive human breast cancer cell nuclei Proteomics 2011 11 159 165 10.1002/pmic.201000344 21182203

[B42] Charpentier AH Bednarek AK Daniel RL Hawkins KA Laflin KJ Gaddis S MacLeod MC Aldaz CM Effects of estrogen on global gene expression: identification of novel targets of estrogen action Cancer Res 2000 60 5977 5983 11085516

[B43] Yang S-H Liu R Perez EJ Wen Y Stevens SM Valencia T Brun-Zinkernagel A-M Prokai L Will Y Dykens J Mitochondrial localization of estrogen receptor {beta} PNAS 2004 101 4130 4135 10.1073/pnas.0306948101 15024130 PMC384706

[B44] Simpkins JW Yang S-H Sarkar SN Pearce V Estrogen actions on mitochondria--Physiological and pathological implications Molecular and Cellular Endocrinology 2008 290 51 59 10.1016/j.mce.2008.04.013 18571833 PMC2737506

[B45] Yang S-H Sarkar SN Liu R Perez EJ Wang X Wen Y Yan L-J Simpkins JW Estrogen Receptor {beta} as a Mitochondrial Vulnerability Factor J Biol Chem 2009 284 9540 9548 10.1074/jbc.M808246200 19189968 PMC2666606

[B46] Simpkins JW Yi KD Yang S-H Dykens JA Mitochondrial mechanisms of estrogen neuroprotection Biochimica et Biophysica Acta (BBA)-General Subjects 2010 1800 1113 1120 10.1016/j.bbagen.2009.11.013 PMC2889195 19931595

[B47] Katzenellenbogen BS Norman MJ Eckert RL Peltz SW Mangel WF Bioactivities, estrogen receptor interactions, and plasminogen activator- inducing activities of tamoxifen and hydroxytamoxifen isomers in MCF-7 human breast cancer cells Cancer Res 1984 44 112 119 6537799

[B48] Skog S He Q Khoshnoud R Fornander T Rutqvist LE Genes related to growth regulation, DNA repair and apoptosis in an oestrogen receptor-negative (MDA-231) versus an oestrogen receptor-positive (MCF-7) breast tumour cell line Tumour Biol 2004 25 41 47 10.1159/000077722 15192311

[B49] Durant ST Nickoloff JA Good timing in the cell cycle for precise DNA repair by BRCA1 Cell Cycle 2005 4 1216 1222 10.4161/cc.4.9.2027 16103751

[B50] Wang CLA Coluccio LM Kwang WJ New Insights into the Regulation of the Actin Cytoskeleton by Tropomyosin International Review of Cell and Molecular Biology 2010 281 Academic Press 91 128 20460184 10.1016/S1937-6448(10)81003-2 PMC2923581

[B51] Pinton G Thomas W Bellini P Manente AG Favoni RE Harvey BJ Mutti L Moro L Estrogen receptor beta exerts tumor repressive functions in human malignant pleural mesothelioma via EGFR inactivation and affects response to gefitinib PLoS One 5 e14110 10.1371/journal.pone.0014110 PMC2993924 21124760

[B52] Fan P Wang J Santen RJ Yue W Long-term Treatment with Tamoxifen Facilitates Translocation of Estrogen Receptor {alpha} out of the Nucleus and Enhances its Interaction with EGFR in MCF-7 Breast Cancer Cells Cancer Res 2007 67 1352 1360 10.1158/0008-5472.CAN-06-1020 17283173

[B53] Picard N Charbonneau C Sanchez M Licznar A Busson M Lazennec G Tremblay A Phosphorylation of Activation Function-1 Regulates Proteasome-Dependent Nuclear Mobility and E6-Associated Protein Ubiquitin Ligase Recruitment to the Estrogen Receptor {beta} Mol Endocrinol 2008 22 317 330 17962381 10.1210/me.2007-0281 PMC2346536

[B54] Ladanyi M Pao W Lung adenocarcinoma: guiding EGFR-targeted therapy and beyond Mod Pathol 2008 21 Suppl 2 S16 22 18437168 10.1038/modpathol.3801018

[B55] Lo HW Nuclear mode of the EGFR signaling network: biology, prognostic value, and therapeutic implications Discov Med 10 44 51 PMC3637667 20670598

[B56] Marquez DC Lee J Lin T Pietras RJ Epidermal growth factor receptor and tyrosine phosphorylation of estrogen receptor Endocrine 2001 16 73 81 10.1385/ENDO:16:2:073 11887937

[B57] Konishi A Berk BC Epidermal Growth Factor Receptor Transactivation Is Regulated by Glucose in Vascular Smooth Muscle Cells Journal of Biological Chemistry 2003 278 35049 35056 10.1074/jbc.M304913200 12829718

[B58] Damstrup L Wandahl Pedersen M Bastholm L Elling F Skovgaard Poulsen H Epidermal growth factor receptor mutation type III transfected into a small cell lung cancer cell line is predominantly localized at the cell surface and enhances the malignant phenotype Int J Cancer 2002 97 7 14 10.1002/ijc.1572 11774237

[B59] Kuan CT Wikstrand CJ Bigner DD EGF mutant receptor vIII as a molecular target in cancer therapy Endocr Relat Cancer 2001 8 83 96 10.1677/erc.0.0080083 11397666

[B60] Sengupta P Bosis E Nachliel E Gutman M Smith SO Mihályné G Zaitseva I McLaughlin S EGFR Juxtamembrane Domain, Membranes, and Calmodulin: Kinetics of Their Interaction Biophysical Journal 2009 96 4887 4895 10.1016/j.bpj.2009.03.027 19527647 PMC2712057

[B61] Li C Iida M Dunn EF Ghia AJ Wheeler DL Nuclear EGFR contributes to acquired resistance to cetuximab Oncogene 2009 28 3801 3813 10.1038/onc.2009.234 19684613 PMC2900381

[B62] Armour AA Watkins CL The challenge of targeting EGFR: experience with gefitinib in nonsmall cell lung cancer Eur Respir Rev 2010 19 186 196 10.1183/09059180.00005110 20956191 PMC9487283

[B63] Choi SH Mendrola JM Lemmon MA EGF-independent activation of cell-surface EGF receptors harboring mutations found in gefitinib-sensitive lung cancer Oncogene 2007 26 1567 1576 10.1038/sj.onc.1209957 16953218

[B64] Tornaletti S DNA repair in mammalian cells: Transcription-coupled DNA repair: directing your effort where it's most needed Cell Mol Life Sci 2009 66 1010 1020 10.1007/s00018-009-8738-x 19153656 PMC11131556

[B65] Hanawalt PC Spivak G Transcription-coupled DNA repair: two decades of progress and surprises Nat Rev Mol Cell Biol 2008 9 958 970 10.1038/nrm2549 19023283

[B66] Zheng L Annab LA Afshari CA Lee WH Boyer TG BRCA1 mediates ligand-independent transcriptional repression of the estrogen receptor Proc Natl Acad Sci USA 2001 98 9587 9592 10.1073/pnas.171174298 11493692 PMC55496

[B67] Ma YX Tomita Y Fan S Wu K Tong Y Zhao Z Song LN Goldberg ID Rosen EM Structural determinants of the BRCA1: estrogen receptor interaction Oncogene 2005 24 1831 1846 10.1038/sj.onc.1208190 15674350

[B68] Planchard D Loriot Y Goubar A Commo F Soria JC Differential expression of biomarkers in men and women Semin Oncol 2009 36 553 565 10.1053/j.seminoncol.2009.09.004 19995647

[B69] Murphy CG Moynahan ME BRCA gene structure and function in tumor suppression: a repair-centric perspective Cancer J 2010 16 39 47 10.1097/PPO.0b013e3181cf0204 20164689

[B70] Venkitaraman AR Linking the cellular functions of BRCA genes to cancer pathogenesis and treatment Annu Rev Pathol 2009 4 461 487 10.1146/annurev.pathol.3.121806.151422 18954285

[B71] Ma Y Fan S Hu C Meng Q Fuqua SA Pestell RG Tomita YA Rosen EM BRCA1 Regulates Acetylation and Ubiquitination of Estrogen Receptor-{alpha} Mol Endocrinol 2010 24 76 90 10.1210/me.2009-0218 19887647 PMC2802901

