# Supplementary material for: Ligand-dependent differences in estrogen receptor beta-interacting proteins identified in lung adenocarcinoma cells corresponds to estrogenic responses
Source: Proteome Sci. 2011 Sep 27;9:60. doi: 10.1186/1477-5956-9-60 (PMC3192725; doi:10.1186/1477-5956-9-60)
Supplement: Additional file 1 — Supplemental Figure 1: Experimental design for identification of ERβ-FLAG interacting proteins in transfected A549 and H1793 lung adenocarcinoma cells. A549 and H1793 cells were incubated in phenol red-free medium with 5% DCC-stripped serum for 3 days prior to 1 h treatment with ethanol (EtOH, 1:1,000 dilution) or 10 nM E2. WCE (1 mg) from H1793 and A549 was preincubated with or without 355 fmol rhFLAG-ERβ for 1 h at 4°C and added to EZview™Red ANTI- FLAG-M2 affinity beads (Sigma) followed by overnight incubation at 4°C with rotation. After rinsing, as indicated, proteins were eluted with 6 M urea and digested with trypsin prior to LC-MS/MS analysis described in Materials and Methods. In parallel, samples of eluted proteins were separated by SDS PAGE gels and were stained with silver or were transferred for western blot. These western blot images demonstrate ERβ-protein capture. Ingenuity Pathway Analysis (IPA) was used to identify defined canonical pathways and functional classifications of the identified ERβ-interacting proteins. [file 1477-5956-9-60-S1.PPT]

## Slide 1
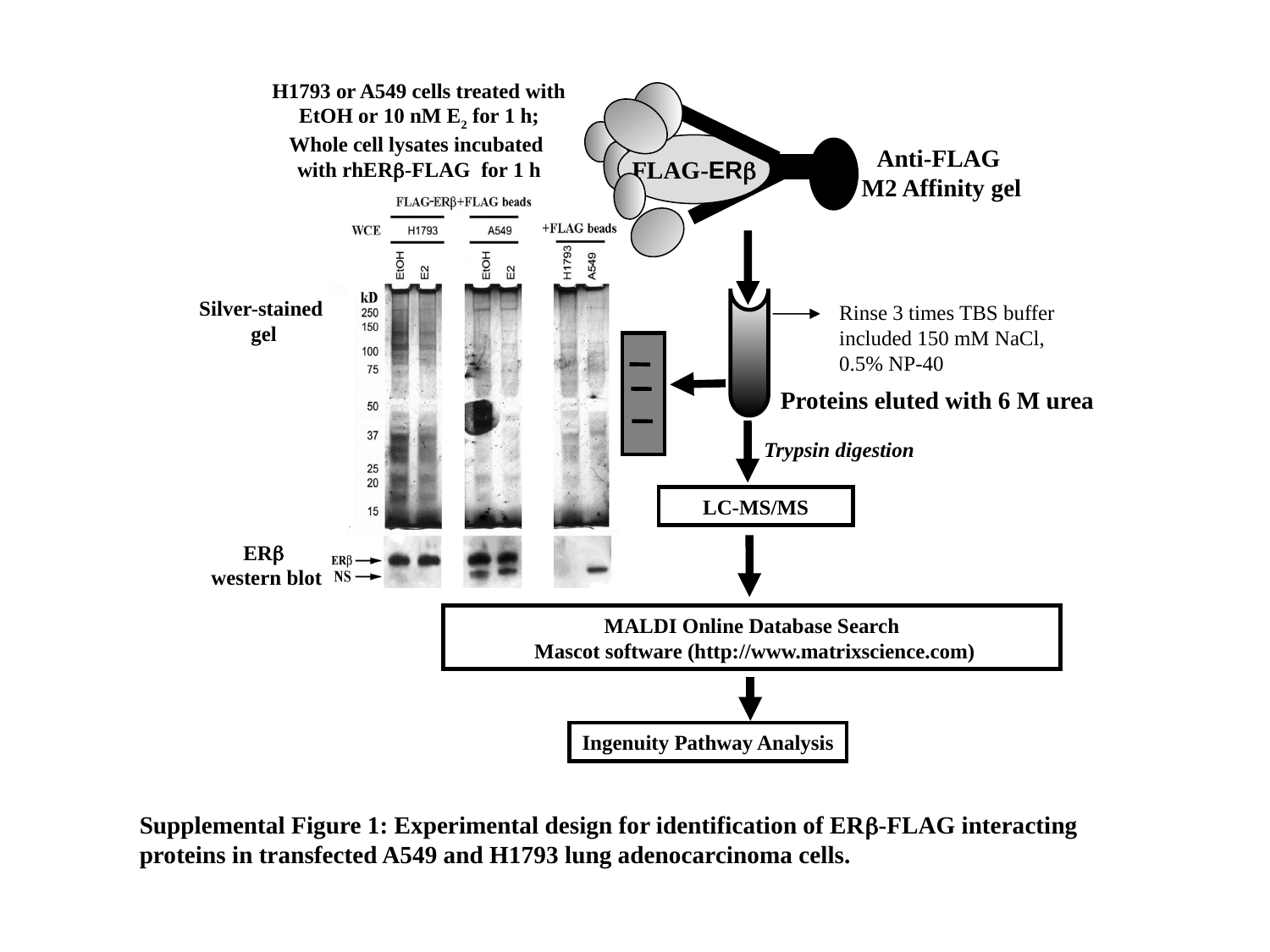

H1793 or A549 cells treated with
EtOH or 10 nM E2 for 1 h;
Whole cell lysates incubated
with rhERβ-FLAG for 1 h
FLAG-ERβ
Anti-FLAG
 M2 Affinity gel
Silver-stained
 gel
Rinse 3 times TBS buffer
included 150 mM NaCl,
0.5% NP-40
SDS-PAGE
Proteins eluted with 6 M urea
Trypsin digestion
LC-MS/MS
ERβ
western blot
MALDI Online Database Search
 Mascot software (http://www.matrixscience.com)
Ingenuity Pathway Analysis
Supplemental Figure 1: Experimental design for identification of ERβ-FLAG interacting proteins in transfected A549 and H1793 lung adenocarcinoma cells.
