# Supplementary figures and images for: Ligand-dependent differences in estrogen receptor beta-interacting proteins identified in lung adenocarcinoma cells corresponds to estrogenic responses
Source: Proteome Sci. 2011 Sep 27;9:60. doi: 10.1186/1477-5956-9-60 (PMC3192725; doi:10.1186/1477-5956-9-60)

## Slide 1
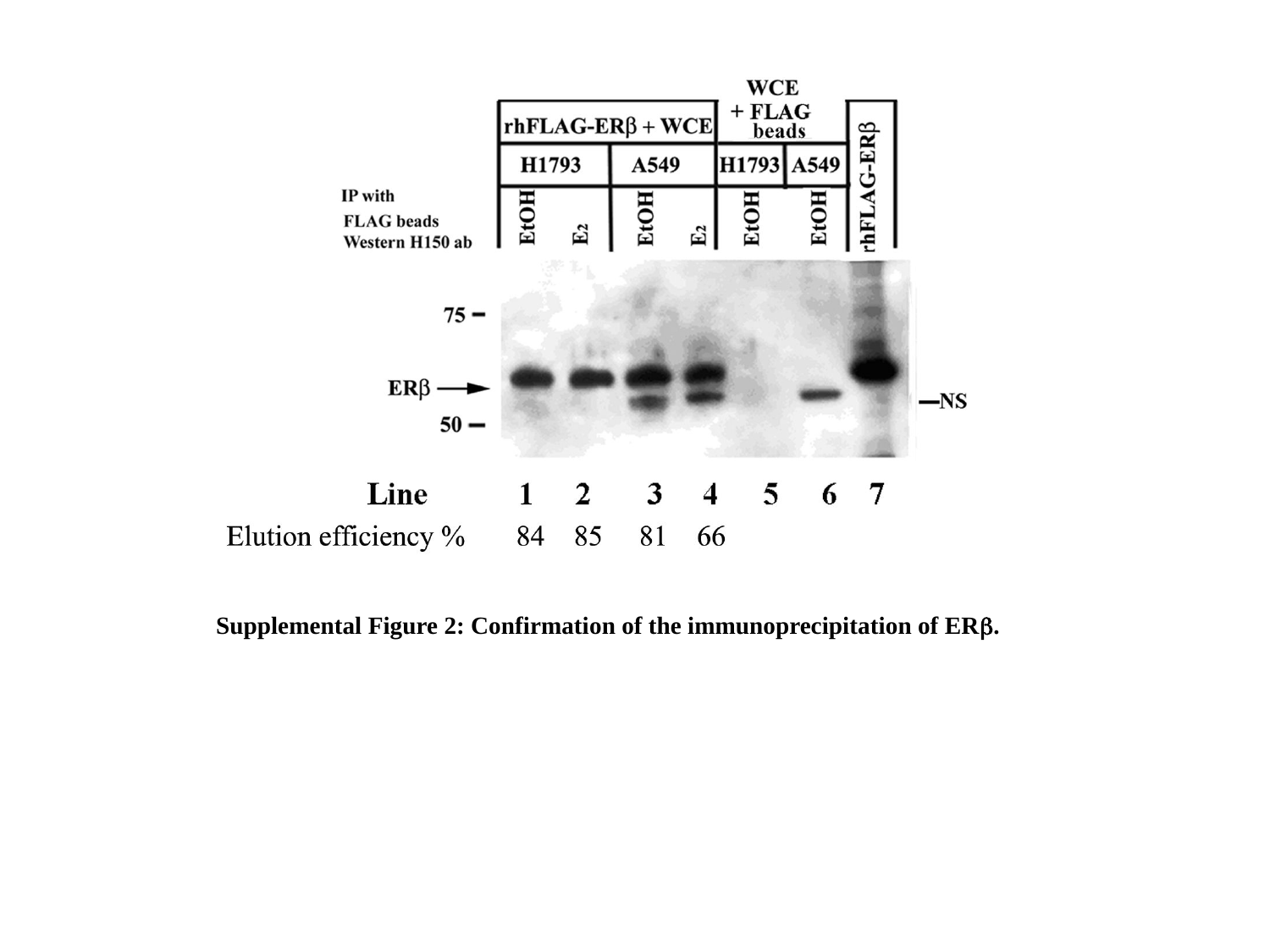

Supplemental Figure 2: Confirmation of the immunoprecipitation of ERβ.

Supplement: Additional file 2 — Supplemental Figure 2: Confirmation of the immunoprecipitation of ERβ. WCE prepared from EtOH or E2- treated H1793 and A549 cells were incubated with FLAG-ERβ as described in Materials and Methods. FLAG-ERβ and interacting proteins were immunoprecipitated using Anti-FLAG M2 affinity beads (Lanes 1-4) and after elution 10 μl of the 100 μl samples was loaded. As a negative control, WCE were incubated with the FLAG beads (Lanes 5-6). Lane 7 was 35.5 fmol rhFLAG-ERβ. The blot was probed with ERβ (H150) antibody. A band at 59 kDa corresponding to ERβ was identified in the IP of H1793 and A549 cell lysates incubated with purified rhFLAG-ERβ protein but not in H1793 or A549 cell extracts incubated with FLAG beads without added rhFLAG-ERβ protein, demonstrating the specificity of the immunocapture for FLAG-ERβ. A nonspecific band of 50 kDa (NS) that was recognized by the ERβ antibody was bound by the FLAG beads in the A549 cells. This may be a splice variant of ERβ. The efficiency of eluting rh-FLAG-ERβ from beads was evaluated by counting the integrated optical densities (IOD) by Un-Scan-It (Silk Scientific, Orem, UT, USA). IODs bands of interest were divided to the control 35.5 fmol rhFLAG-ERβ and counted as %. [file 1477-5956-9-60-S2.PPT]

## Slide 1
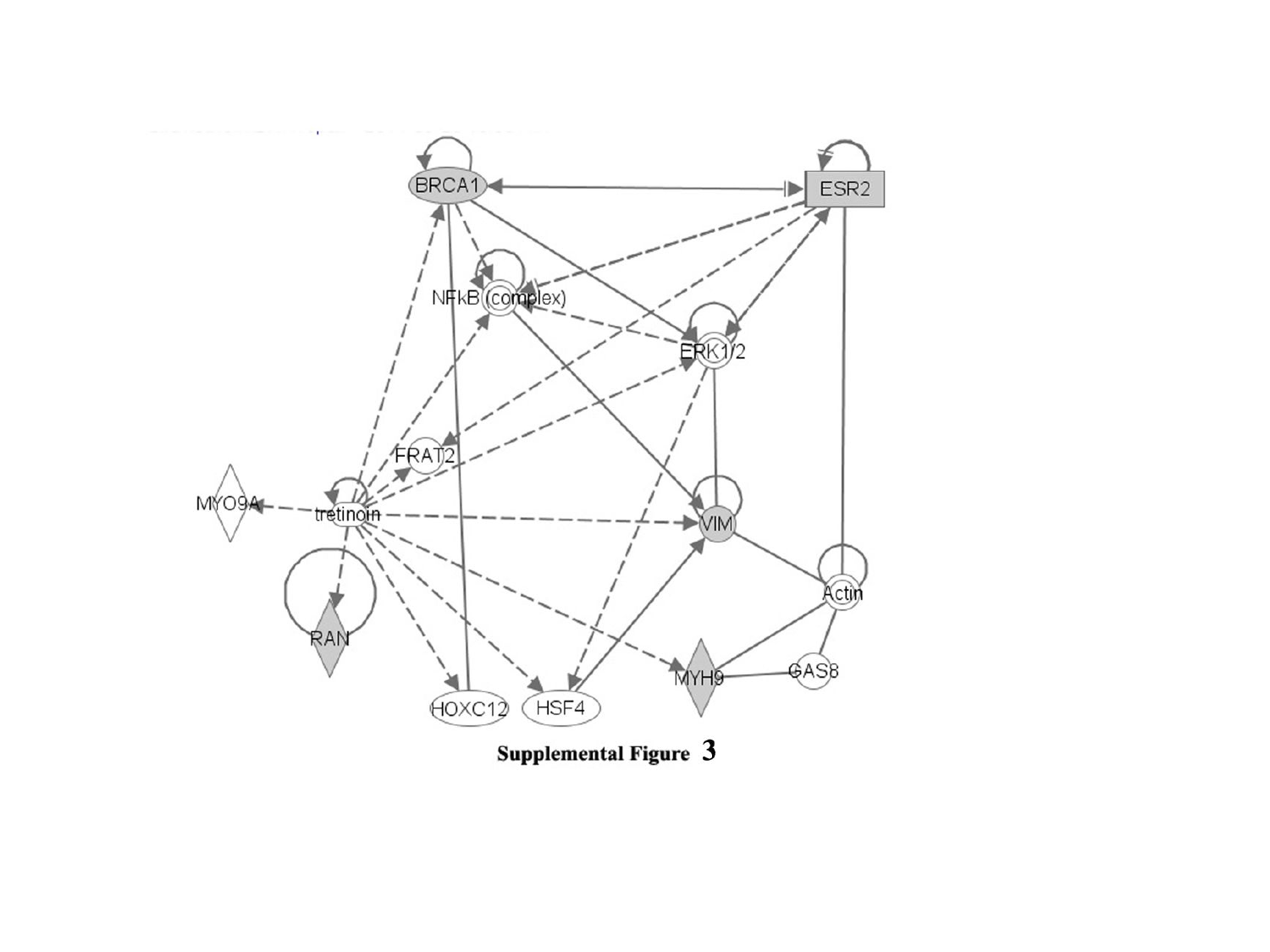

Supplement: Additional file 7 — Supplemental Figure 3: "DNA replication, recombination and repair" network of ERβ-interacting proteins identified in LS-MS/MS. Proteins shaded in grey were identified as ERβ-interacting proteins. Proteins in white are those identified by Ingenuity Knowledge Base. The shapes denote the molecular class of the protein (◇enzyme, ▬ ligand-dependent nuclear receptor, ● other, double circle-group, hexagone-translational regulator). Solid lines indicate direct molecular interaction and dashed lines indicate indirect molecular interaction. [file 1477-5956-9-60-S7.PPT]

## Slide 1
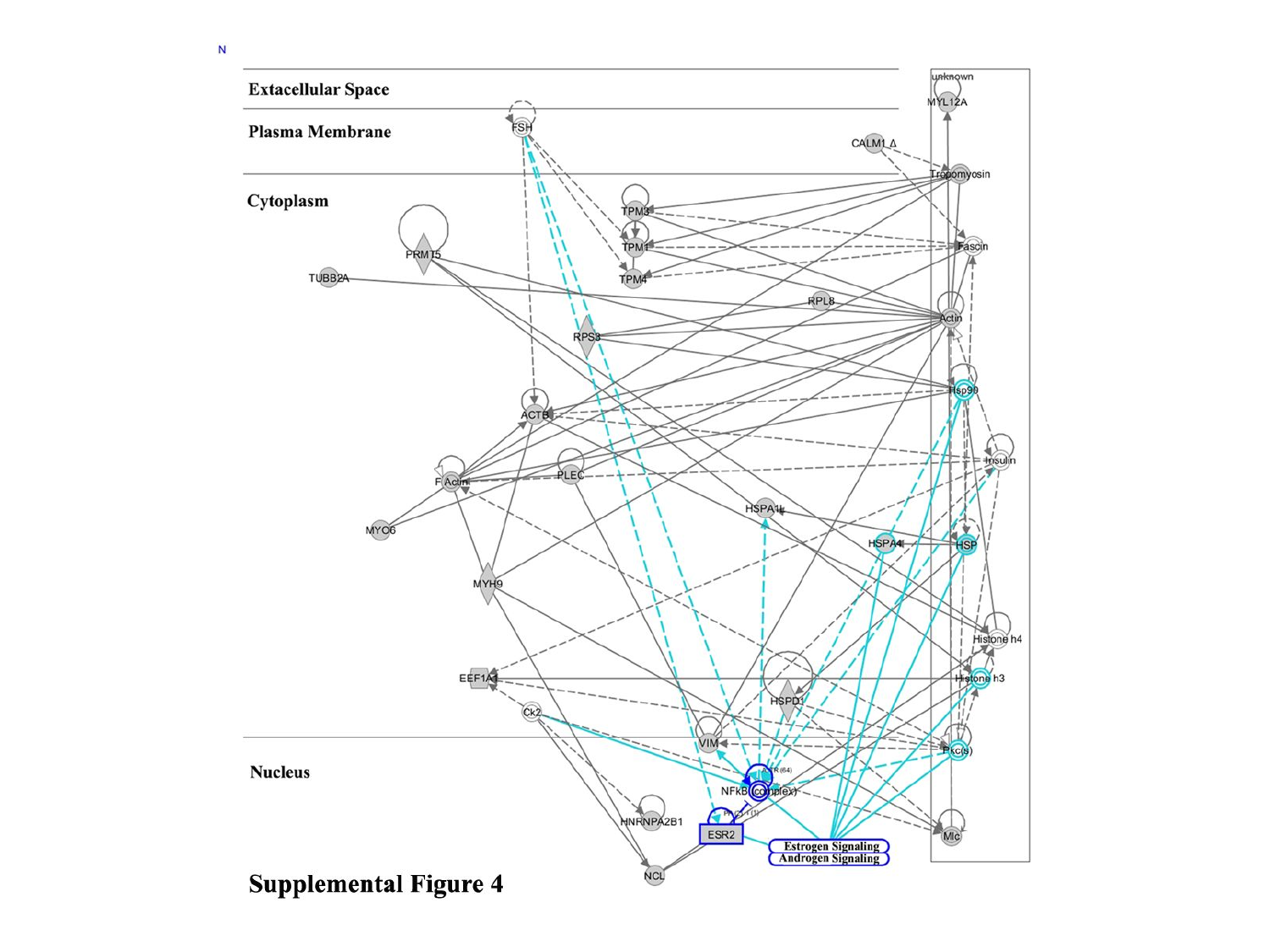

Supplement: Additional file 8 — Supplemental Figure 4: Network pathway analysis of total ERβ-interacting proteins identified in LS-MS/MS. Proteins shaded in grey were identified as ERβ-interacting proteins. Proteins in white are those identified by Ingenuity Knowledge Base. The shapes denote the molecular class of the protein (◇enzyme, ▬ ligand-dependent nuclear receptor, ● other, double circle-group, hexagone-translational regulator). Solid lines indicate direct molecular interaction and dashed lines indicate indirect molecular interaction. [file 1477-5956-9-60-S8.PPT]

## Slide 1
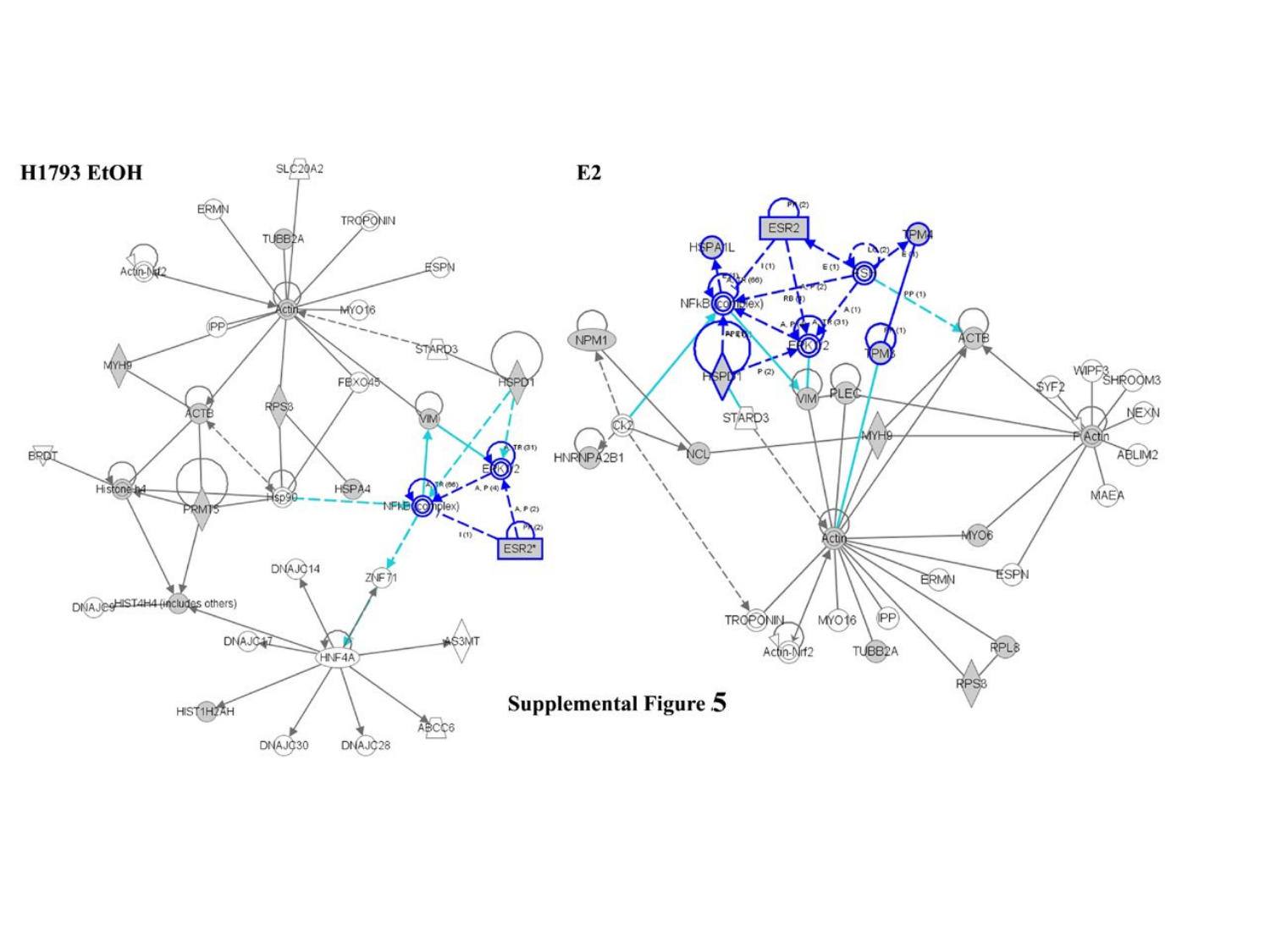

Supplement: Additional file 9 — Supplemental Figure 5: Network pathway analysis of ERβ-interacting proteins in EtOH-(A) and E2- (B) treated H1793 cell lines identified by LC-MS/MS. Proteins shaded in grey were identified as ERβ-interacting proteins. Proteins in white are those identified by Ingenuity Knowledge Base. The shapes denote the molecular class of the protein (◇enzyme, ▬ ligand-dependent nuclear receptor, ● other, double circle-group, hexagone-translational regulator) (Table 2). Solid lines indicate direct molecular interaction, dashed lines indicate indirect molecular interaction and blue lines indicate the proteins discussed in the text. [file 1477-5956-9-60-S9.PPT]

## Slide 1
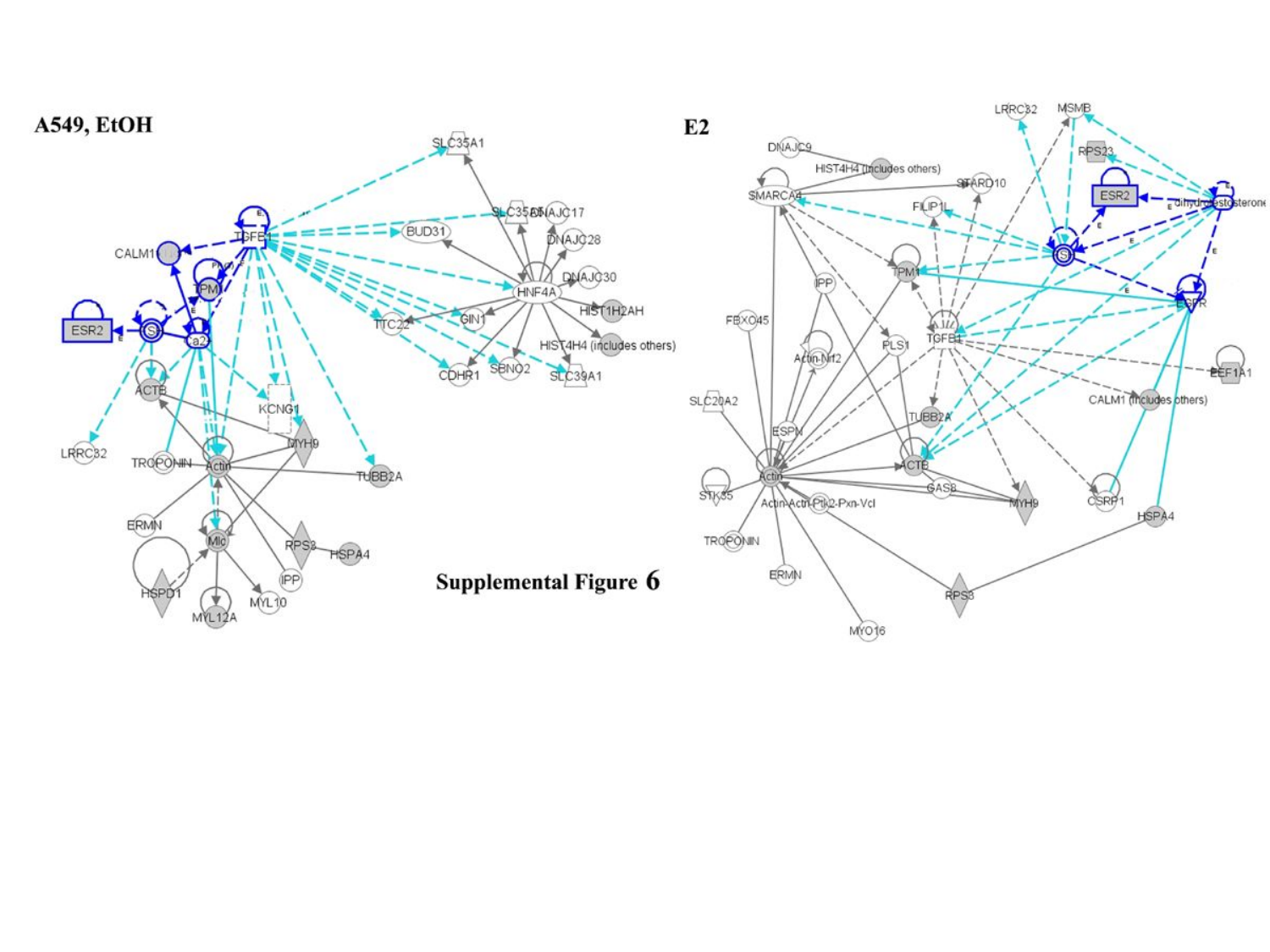

Supplement: Additional file 10 — Supplemental Figure 6: Network pathway analysis of ERβ-interacting proteins in EtOH (A) and E2 (B) treated A549 cell lines identified by LC-MS/MS. Proteins shaded in grey were identified as ERβ-interacting proteins. Proteins in white are those identified by Ingenuity Knowledge Base. The shapes denote the molecular class of the protein (◇enzyme, ▬ ligand-dependent nuclear receptor, ● other, double circle-group, hexagone-translational regulator) (Table 2). Solid lines indicate direct molecular interaction, dashed lines indicate indirect molecular interaction and blue lines indicate the proteins discussed in the text. [file 1477-5956-9-60-S10.PPT]

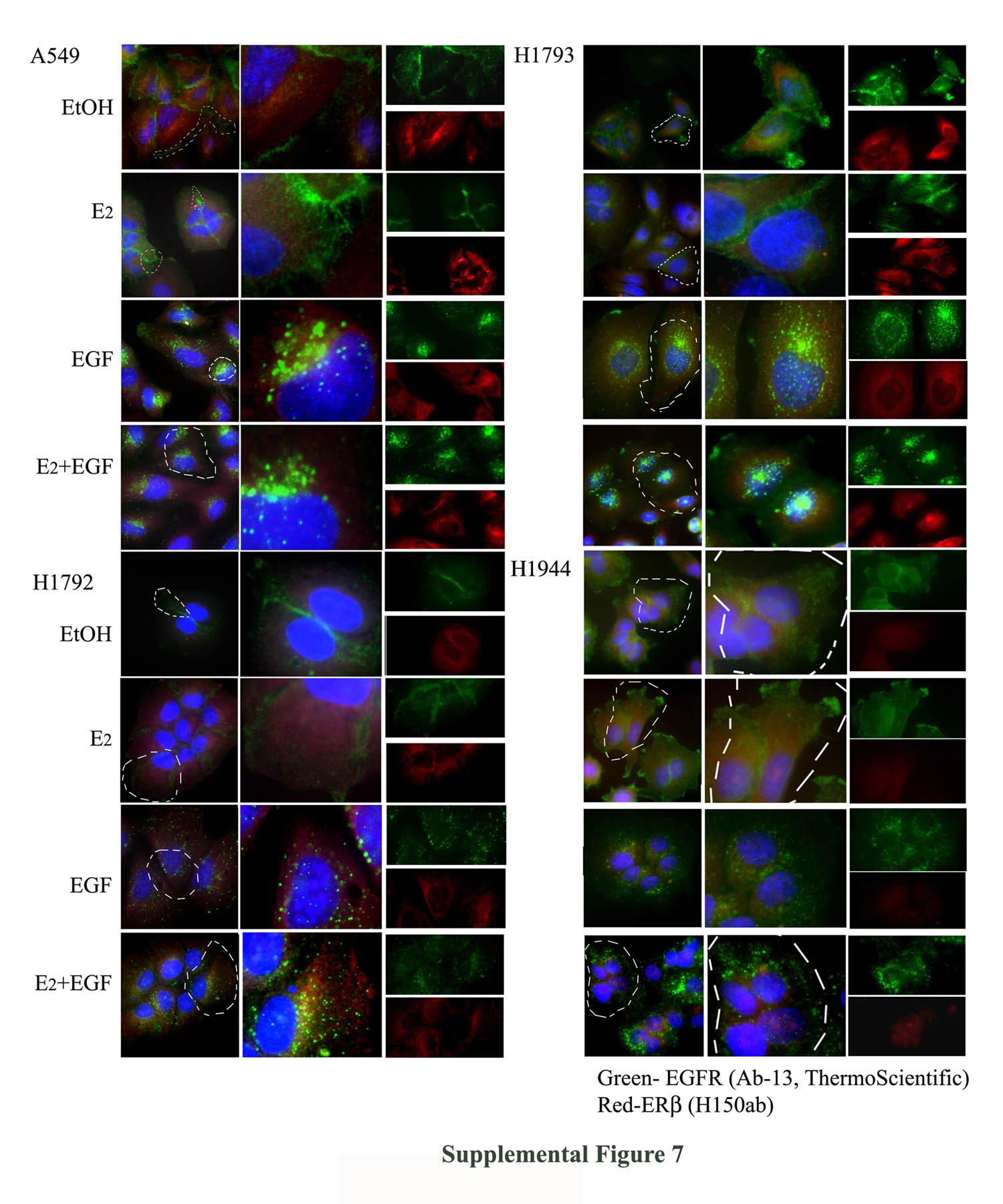

Supplement: Additional file 11 — Supplemental Figure 7: Subcellular localization of EGFR and ERβ in lung adenocarcinoma cells. The indicated lung adenocarcinoma cell lines were treated with EtOH, E2, EGF, or E2+EGF- for 6 h. Merged images for EGFR and ERβ immunocytochemical staining are shown with anti-mouse EGFR Ab-13 (green) and anti-rabbit ERβ ab (06-629) (red). Cells were counterstained with DAPI (blue). At the far right of each panel are non-merged images for EGFR (green) and ERβ (red). Dotted lines outline the cell areas enlarged in the middle panel. [file 1477-5956-9-60-S11.TIFF]
