# Supplementary material for: Ligand-dependent differences in estrogen receptor beta-interacting proteins identified in lung adenocarcinoma cells corresponds to estrogenic responses
Source: Proteome Sci. 2011 Sep 27;9:60. doi: 10.1186/1477-5956-9-60 (PMC3192725; doi:10.1186/1477-5956-9-60)
Supplement: Additional file 4 — Supplemental Table 2: Identification of ERβ interacting proteins in H1793 and A549 cells by LC-MS/MS. This table lists proteins identified as interacting with ERβ in H1793 and A549 lung adenocarcinoma cells treated with EtOH or E2. [file 1477-5956-9-60-S4.DOC]

**Supplemental Table 2: Identification of ERbeta interacting proteins in H1793 and A549 cells by LC-MS/MS.** H1793 and A549 cells were treated for 1 h with EtOH (vehicle) or 10 nM E_2_. WCE from the H1793 and A549 cells were incubated with rhFLAG-ERβ and anti-FLAG M2 affinity gel (Additional file 1, Figure S1 1). ERβ-interacting proteins were eluted with 6 M urea, digested with trypsin, and subjected to LC-MS/MS peptide identification. Columns are self-explanatory: peptide-spectrum matches (PSMs), AAs (amino acid coverage), Score is the sum of all the scores of the individual peptides.

|  | Protein name | Accession | Coverage | #PSMs | #Peptides | #AAs | MW (kDa) | Calc. pI | Score |
| --- | --- | --- | --- | --- | --- | --- | --- | --- | --- |
| **H1793, EtOH** | | | | | | | | | |
| 1 | Tubulin beta-2A chain | Q13885 | 21.80 | 24 | 8 | 445 | 49.9 | 4.89 | 79.48 |
| 2 | Myosin-9] | P35579 | 4.95 | 12 | 7 | 1960 | 226.4 | 5.60 | 40.99 |
| 3 | Actin, cytoplasmic 1 | P60709 | 20.53 | 10 | 5 | 375 | 41.7 | 5.48 | 39.02 |
| 4 | Tubulin alpha-3C/D chain | Q13748 | 20.00 | 11 | 6 | 450 | 49.9 | 5.10 | 33.36 |
| 5 | Vimentin | P08670 | 12.23 | 6 | 6 | 466 | 53.6 | 5.12 | 26.00 |
| 6 | Estrogen receptor beta | Q92731 | 8.11 | 4 | 3 | 530 | 59.2 | 8.47 | 23.54 |
| 7 | Heat shock 70 kDa protein | P08418 | 11.77 | 8 | 5 | 637 | 69.8 | 5.58 | 22.55 |
| 8 | Histone H2A type 1-H | Q96KK5 | 21.88 | 6 | 2 | 128 | 13.9 | 10.89 | 17.66 |
| 9 | 60 kDa heat shock protein, mitochondrial | P10809 | 4.89 | 3 | 3 | 573 | 61.0 | 5.87 | 15.11 |
| 10 | Putative annexin A2-like protein | A6NMY6 | 6.19 | 4 | 2 | 339 | 38.6 | 6.95 | 11.42 |
| 11 | 40S ribosomal protein S3 | P23396 | 13.99 | 4 | 3 | 243 | 26.7 | 9.66 | 11.04 |
| 12 | Protein arginine N-methyltransferase 5 | O14744 | 3.14 | 2 | 2 | 637 | 72.6 | 6.29 | 9.55 |
| 13 | Calmodulin | P62158 | 18.79 | 3 | 2 | 149 | 16.8 | 4.22 | 8.93 |
| 14 | Histone H4 | P62805 | 21.36 | 2 | 2 | 103 | 11.4 | 11.36 | 6.38 |
| 15 | GTP-binding nuclear protein | P62826 | 9.72 | 2 | 2 | 216 | 24.4 | 7.49 | 3.99 |
| **H1793, E_2_** | | | | | | | | | |
| 1 | Actin, cytoplasmic 1 | P60709 | 33.33 | 22 | 8 | 375 | 41.7 | 5.48 | 69.11 |
| 2 | Myosin-9 | P35579 | 6.53 | 15 | 10 | 1960 | 226.4 | 5.60 | 53.87 |
| 3 | Tubulin beta-2A chain | Q13885 | 20.45 | 12 | 7 | 445 | 49.9 | 4.89 | 37.80 |
| 4 | Tubulin alpha-3C/D chain | Q13748 | 19.78 | 9 | 5 | 450 | 49.9 | 5.10 | 29.26 |
| 5 | Tropomyosin alpha-4 chain | P67936 | 14.11 | 8 | 4 | 248 | 28.5 | 4.69 | 25.15 |
| 6 | 60 kDa heat shock protein, mitochondrial | P10809 | 12.22 | 6 | 5 | 573 | 61.0 | 5.87 | 19.84 |
| 7 | Histone H2A type 1-H | Q96KK5 | 27.34 | 6 | 3 | 128 | 13.9 | 10.89 | 16.87 |
| 8 | Heat shock 70 kDa protein 1-like | P34931 | 6.55 | 6 | 3 | 641 | 70.3 | 6.02 | 16.14 |
| 9 | Vimentin | P08670 | 6.22 | 5 | 3 | 466 | 53.6 | 5.12 | 13.52 |
| 10 | Nucleolin | P19338 | 6.90 | 5 | 3 | 710 | 76.6 | 4.70 | 12.31 |
| 11 | Tropomyosin alpha-3 chain | P06753 | 8.10 | 3 | 2 | 284 | 32.8 | 4.72 | 11.98 |
| 12 | Nucleophosmin | P06748 | 14.97 | 4 | 3 | 294 | 32.6 | 4.78 | 11.41 |
| 13 | Myosin-VI | Q9UM54 | 1.78 | 4 | 2 | 1294 | 149.6 | 8.53 | 11.29 |
| 14 | Plectin | Q15149 | 0.51 | 2 | 2 | 4684 | 531.5 | 5.96 | 10.94 |
| 15 | 40S ribosomal protein S3 | P23396 | 10.29 | 4 | 2 | 243 | 26.7 | 9.66 | 9.58 |
| 16 | 60S ribosomal protein | P62917 | 7.00 | 2 | 2 | 257 | 28.0 | 11.03 | 7.45 |
| 17 | Heterogeneous nuclear ribonucleoproteins A2/B1 | P22626 | 5.10 | 3 | 2 | 353 | 37.4 | 8.95 | 6.75 |
| **A549, EtOH** | | | | | | | | | |
| 1 | Tubulin beta-2A chain | Q13885 | 26.29 | 16 | 9 | 445 | 49.9 | 4.89 | 54.24 |
| 2 | Actin, cytoplasmic 1 | P60709 | 21.87 | 9 | 5 | 375 | 41.7 | 5.48 | 32.10 |
| 3 | Tubulin alpha-3C/D chain | Q13748 | 25.11 | 11 | 7 | 450 | 49.9 | 5.10 | 29.68 |
| 4 | Heat shock 70 kDa protein homolog | P08418 | 8.01 | 7 | 4 | 637 | 69.8 | 5.58 | 24.45 |
| 5 | 60 kDa heat shock protein, mitochondrial | P10809 | 7.85 | 6 | 4 | 573 | 61.0 | 5.87 | 19.57 |
| 6 | Histone H2A type 1-H | Q96KK5 | 21.88 | 6 | 2 | 128 | 13.9 | 10.89 | 17.25 |
| 7 | 40S ribosomal protein S3 | P23396 | 13.99 | 5 | 3 | 243 | 26.7 | 9.66 | 12.14 |
| 8 | Histone H4 | P62805 | 29.13 | 4 | 3 | 103 | 11.4 | 11.36 | 12.10 |
| 9 | Tropomyosin alpha-1 chain | P09493 | 8.10 | 2 | 2 | 284 | 32.7 | 4.74 | 9.89 |
| 10 | Myosin-9 | P35579 | 1.33 | 2 | 2 | 1960 | 226.4 | 5.60 | 8.31 |
| 11 | Calmodulin | P62158 | 18.79 | 3 | 2 | 149 | 16.8 | 4.22 | 6.68 |
| 12 | Myosin regulatory light chain 12A | P19105 | 12.28 | 2 | 2 | 171 | 19.8 | 4.81 | 6.12 |
| **A549, E_2_** | | | | | | | | | |
| 1 | Tubulin beta-2A chain | Q13885 | 24.94 | 17 | 9 | 445 | 49.9 | 4.89 | 58.84 |
| 2 | Actin, cytoplasmic | P60709 | 26.13 | 9 | 6 | 375 | 41.7 | 5.48 | 33.75 |
| 3 | Tubulin alpha-3C/D chain | Q13748 | 20.00 | 8 | 6 | 450 | 49.9 | 5.10 | 24.54 |
| 4 | Heat shock 70 kDa protein homolog | P08418 | 8.01 | 7 | 4 | 637 | 69.8 | 5.58 | 18.90 |
| 5 | Myosin-9 | P35579 | 2.04 | 5 | 3 | 1960 | 226.4 | 5.60 | 18.70 |
| 6 | 40S ribosomal protein S3 | P23396 | 13.99 | 6 | 3 | 243 | 26.7 | 9.66 | 14.00 |
| 7 | 60 kDa heat shock protein, mitochondrial | P10809 | 7.85 | 5 | 4 | 573 | 61.0 | 5.87 | 13.05 |
| 8 | Histone H4 | P62805 | 33.01 | 4 | 3 | 103 | 11.4 | 11.36 | 12.11 |
| 9 | Elongation factor 1-alpha 1 | P68104 | 6.28 | 3 | 3 | 462 | 50.1 | 9.01 | 11.64 |
| 10 | Calmodulin | P62158 | 18.79 | 3 | 2 | 149 | 16.8 | 4.22 | 8.47 |
| 11 | Tropomyosin alpha-1 chain | P09493 | 7.04 | 2 | 2 | 284 | 32.7 | 4.74 | 7.67 |
| 12 | 40S ribosomal protein S23 | P62266 | 16.08 | 2 | 2 | 143 | 15.8 | 10.49 | 5.21 |
