# Supplementary material for: Ligand-dependent differences in estrogen receptor beta-interacting proteins identified in lung adenocarcinoma cells corresponds to estrogenic responses
Source: Proteome Sci. 2011 Sep 27;9:60. doi: 10.1186/1477-5956-9-60 (PMC3192725; doi:10.1186/1477-5956-9-60)
Supplement: Additional file 5 — Supplemental Table 3: Identification of ERβ-interacting proteins in 4-hydroxytamoxifen (4-OHT) treated H1793 by LC-MS/MS. This table lists proteins identified as interacting with ERβ in H1793 lung adenocarcinoma cells treated with 100 nM 4-OHT. [file 1477-5956-9-60-S5.DOC]

**Supplemental Table 3: Identification of ERβ-interacting proteins in 4-hydroxytamoxifen (4-OHT)- treated H1793 human lung adenocarcinoma cells by LC-MS/MS.** H1793 cells were treated for 1 h with 100 nM 4-OHT. WCE were incubated with rhFLAG-ERβ and anti-FLAG M2 affinity gel (Additional file 1, Fig. S1). ERβ-interacting proteins were eluted with 6 M urea, digested with trypsin, and subjected to LC-MS/MS peptide identification. Columns are self-explanatory: peptide-spectrum matches (PSMs), AAs (amino acid coverage), Score is the sum of all the scores of the individual peptides. Proteins are listed from highest to lowest score. The last four rows indicate identification of these proteins (indicated by +) as interacting with ERβ in EtOH- or E_2_- treated H1793 and A549 cell lines (Additional file 4, Table S2).

|  | Protein name | Accession | Coverage | #PSMs | #Peptides | #AAs | MW (kDa) | Calc. pI | Score | H1793  4-OHT | H1793  EtOH | H1793  E_2_ | A549  EtOH | A549  E_2_ |
| --- | --- | --- | --- | --- | --- | --- | --- | --- | --- | --- | --- | --- | --- | --- |
| 1 | Tubulin beta-2A – TUBB2A | Q13885 | 27.64 | 29 | 9 | 445 | 49.9 | 4.89 | 98.55 | + | + | + | + | + |
| 2 | Heat shock cognate 70 kDa protein – HSPA8 | P11142 | 13.78 | 13 | 7 | 646 | 70.9 | 5.52 | 41.27 | + | + | + | + | + |
| 3 | Tubulin alpha-3C/D TUBA3C | Q13748 | 18.44 | 9 | 5 | 450 | 49.9 | 5.10 | 32.73 | + | + | + | + | + |
| 4 | Estrogen receptor beta ESR2 | P10809 | 4.89 | 3 | 3 | 573 | 61.0 | 5.87 | 15.11 | + |  |  |  |  |
| 5 | Actin, gamma-enteric smooth muscle ACTH | A6NMY6 | 6.19 | 4 | 2 | 339 | 38.6 | 6.95 | 11.42 | + |  |  |  |  |
| 6 | Elongation factor 1-alpha 1 EEF1A1 | P23396 | 13.99 | 4 | 3 | 243 | 26.7 | 9.66 | 11.04 | + |  |  |  | + |
| 7 | 40S ribosomal protein RPS3 | O14744 | 3.14 | 2 | 2 | 637 | 72.6 | 6.29 | 9.55 | + | + | + | + | + |
| 8 | 60S ribosomal protein RL23] | P62158 | 18.79 | 3 | 2 | 149 | 16.8 | 4.22 | 8.93 | + |  | + |  |  |
| 9 | 14-3-3 protein epsilon 1433E | P62805 | 21.36 | 2 | 2 | 103 | 11.4 | 11.36 | 6.38 | + |  |  |  |  |
| 10 | Heat shock protein HSP 90-alpha HS90A | P62826 | 9.72 | 2 | 2 | 216 | 24.4 | 7.49 | 3.99 | + |  |  |  |  |
